# Amino-acid inserts of HIV-1 capsid (CA) induce CA degradation and abrogate viral infectivity: Insights for the dynamics and mechanisms of HIV-1 CA decomposition

**DOI:** 10.1038/s41598-019-46082-2

**Published:** 2019-07-08

**Authors:** Masayuki Amano, Haydar Bulut, Sadahiro Tamiya, Tomofumi Nakamura, Yasuhiro Koh, Hiroaki Mitsuya

**Affiliations:** 1 Department of Hematology, Rheumatology, and Infectious Diseases, Kumamoto University School of Medicine, Kumamoto, 860-8556 Japan; 2 Experimental Retrovirology Section, HIV and AIDS Malignancy Branch, Center for Cancer Research, National Cancer Institute, National Institutes of Health, Bethesda, MD 20892 USA; 3 Department of Refractory Viral Infection, National Center for Global Health and Medicine Research Institute, Tokyo, 162-8655 Japan

**Keywords:** Retrovirus, Virus structures

## Abstract

Accumulation of amino acid (AA) insertions/substitutions are observed in the Gag-protein of HIV-1 variants resistant to HIV-1 protease inhibitors. Here, we found that HIV-1 carrying AA insertions in capsid protein (CA) undergoes aberrant CA degradation. When we generated recombinant HIV-1s (rHIV-1s) containing 19-AAs in Gag, such insertions caused significant CA degradation, which initiated in CA’s C-terminal. Such rHIV-1s had remarkable morphological abnormality, decreased infectivity, and no replicative ability, which correlated with levels of CA degradation. The CA degradation observed was energy-independent and had no association with cellular/viral proteolytic mechanisms, suggesting that the CA degradation occurs due to conformational/structural incompatibility caused by the 19-AA insertions. The incorporation of degradation-prone CA into the wild-type CA resulted in significant disruption of replication competence in “chimeric” virions. The data should allow better understanding of the dynamics and mechanisms of CA decomposition/degradation and retroviral uncoating, which may lead to new approach for antiretroviral modalities.

## Introduction

The currently available combination antiretroviral therapy (cART) for human immunodeficiency virus type 1 (HIV-1) infection has been shown to effectively suppress viral replication and significantly extend the life expectancy of infected individuals^1,2^. However, in the course of life-long treatment, drug-resistant HIV-1 often emerges, which has been a major contributor to treatment failure^3–5^. HIV-1 often develops high levels of cross-resistance against multiple drugs by acquiring a variety of amino acid (AA) substitutions near the active sites of targeted enzymes^6–10^. Such substitutions often compromise the enzymatic functions of the viral reverse transcriptase, protease (PR), and integrase^11–16^. During the development of resistance to certain protease inhibitors (PIs), HIV-1 often acquires, in addition to resistance-associated AA substitutions in PR, “compensatory” AA substitutions in Gag proteins. These compensatory AA substitutions do not directly confer drug-resistance but rather improve the otherwise compromised catalytic functions of the mutated PR^17,18^. Certain AA insertions in the proximity of Gag cleavage sites also restore the otherwise compromised enzymatic activity of PRs, enabling PI-resistant HIV-1 variants to remain replication-competent^19^. However, the replication competence of such HIV-1 variants does not get fully restored, viral replication of multi-PI-resistant variants remain poor compared with that of wild-type HIV-1.

In the present study, we introduced a 19-AA insertion at various positions in the Gag region of infectious HIV-1s using the Tn5-based transposon system and examined their effects on the processing and maturation of the Gag structural protein. Interestingly, we observed the emergence of enhanced CA degradation products, which had not been reported previously, in HIV-1 variants generated by transposon (HIV^TP^s)-containing the 19-AA insertion in the NTD region of CA. Our results also suggested that the observed CA degradation rapidly progressed in time-dependent manner, and CA degradation had no association with cellular/viral proteolytic mechanisms. CA degradation-inducing HIV^TP^s had drastic morphological abnormality, compromised infectivity, and no replicative ability. Furthermore, we show that upon the cleavage of the Gag between the CA and p2-NC, CA degradation occurs and that the CA degradation predominantly occurs in the CTD of the CA.

## Results

### CA degradation observed in HIV-1 variants with AA insertion in the Gag region

We have previously shown that certain AA insertions near the cleavage sites in Gag polyprotein result in the restoration of the otherwise compromised enzymatic activity of mutated PRs, enabling multi-PI-resistant HIV-1 variants to remain replication-competent^19^. We also found that drug-resistant HIV-1 variants carrying multiple (3~6) AA insertions in Gag undergoes aberrant CA degradation in Western blotting (WB) employing virion lysates of each HIV-1 with Gag AA insert produced by transfected COS-7 cells (^19^; Fig. S1a).

**Figure 1:**
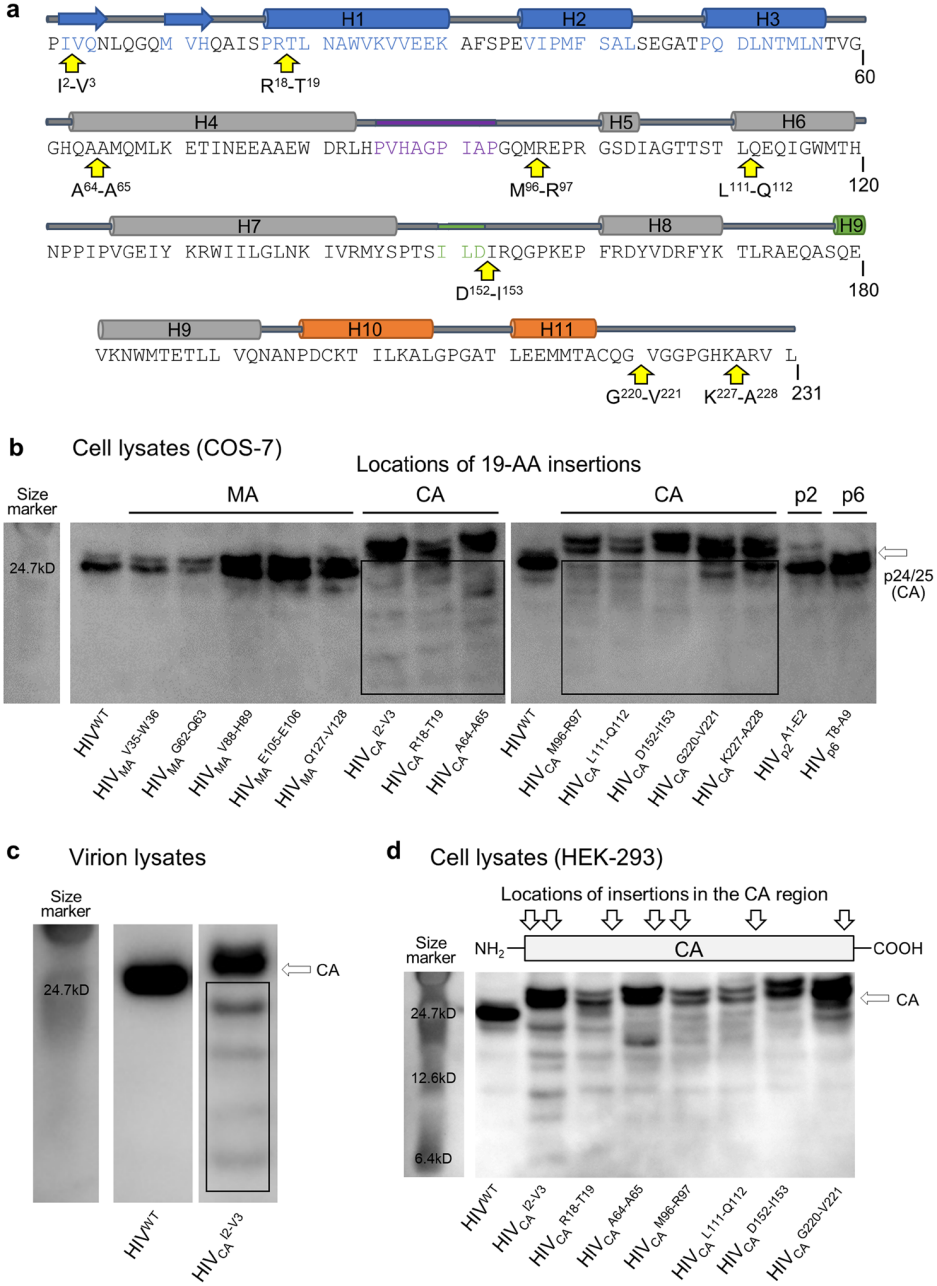
CA degradation observed in HIV-1 variants with the AA insertion in Gag region. **(a)** Locations of each 19-AA insertion in CA of HIV^TP^ clones generated by using the Tn5-based transposon system are shown. The sequence of wild-type CA (CA^WT^) is shown with its secondary structure diagram. HIV-1 CA consists of the N-terminal domain (NTD; residues 1–145) and the C-terminal domain (CTD; residues 151–231) connected by 5 AAs that act as a flexible linker. Each insertion site is indicated by yellow arrow. The hexamer structure is stabilized by interactions formed by s hairpin and first 3 helices H1, H2, H3 as shown in blue. Two- and three-fold interactions take place between hexamer units are shown in green and orange, respectively. Cyclophilin A-binding site is indicated in purple. **(b)** CA degradation in the lysates of COS-7 cells producing various 19-AAs-containing HIV clones (HIV^TP^s). The lysates of COS-7 cells producing HIV^WT^ or HIV^TP^s were examined for enhanced degradation. Each lysate sample was prepared 72 hrs after transfection and subjected to WB with anti-p24 polyclonal anti-serum. The boxes indicate lower-molecular-weight CA degradation products than the mature p24/p25 (CA). Results shown is representative of more than ten independent experiments. **(c)** The CA degradation products are identified in the lysates of HIV_CA_^I2-V3^. The supernatants of COS-7 cells producing HIV^WT^ or HIV_CA_^I2-V3^ were collected 72 hrs after transfection, cleaned with 0.22 μm pore size filters, and subjected to ultracentrifugation (20,000 *g*, 4 °C, 24 hrs). The obtained virions were lysed and was subjected to WB. Results shown is representative of three independent experiments. **(d)** The lysates of HEK-293 cells producing HIV^WT^ or HIV^TP^s showed the similar degradation products as seen in the COS-7 cell lysates as above. All samples in (**b**–**d**) were normalized by their protein concentrations (20 μg/well). Results shown is representative of seven independent experiments. Full-length blots/gels are presented in Fig. S9.

In order to further elucidate the effects of AA insertions on the infectivity and replication capacity of HIV-1, we generated a series of recombinant HIV-1 clones-containing a 19-AA insertion randomly introduced into the Gag region of HIV-1 using the Tn5-based transposon system (HIV^TP^: Figs 1a and S1b). HIV^TP^ we generated included 5 HIV^TP^ species-containing the 19-AA insertion in MA (HIV_MA_^V35-W36^, HIV_MA_^G62-Q63^, HIV_MA_^V88-H89^, HIV_MA_^E105-E106^, and HIV_MA_^Q127-V128^), 8 HIV^TP^ species-containing the insertion in CA (HIV_CA_^I2-V3^, HIV_CA_^R18-T19^, HIV_CA_^A64-A65^, HIV_CA_^M96-R97^, HIV_CA_^L111-Q112^, HIV_CA_^D152-I153^, HIV_CA_^G220-V221^, and HIV_CA_^K227-A228^), one HIV^TP^-containing the insertion in p2 (HIV_p2_^A1-E2^), and one HIV^TP^-containing the insertion in p6 (HIV_p6_^T8-A9^)(Fig. S1b; AA sequences of inserts are described in Table S1). When the lysates of COS-7 cells producing the 19-AAs-containing HIV^TP^ were subjected to WB using an HIV-1 CA-specific Gag polyclonal antibody (pAb)(Fig. 1b), notable levels of CA-specific signals, of which molecular-weights were less than that of CA, were observed. Such signals were predominantly and almost exclusively identified in HIV^TP^ species that contained the insertions in CA. The signals (Fig. 1b, boxed) are identified as ladders consisting of different molecular-weight proteins but not of smear-like proteins. Thus, it is thought that those proteins represent orderly-processed CA degradation products. Moreover, the amounts of CA degradation products appeared to be greater when the inserts were introduced in the N-terminal domain of CA (HIV^TP^-CA^INS^NTD: HIV_CA_^I2-V3^, HIV_CA_^R18-T19^, HIV_CA_^A64-A65^, HIV_CA_^M96-R97^, HIV_CA_^L111-Q112^) than in the C-terminal domain of CA (HIV^TP^-CA^INS^CTD). In contrast, no or least degradation products were observed in the lysates of cells producing HIV^WT^ or HIV^TP^s-containing the insert in MA, p2, or p6 proteins (Fig. 1b). These degradation products profiles strongly suggest that the insertion of the 19-exogenous-AA in CA caused the enhanced CA degradation. When we obtained condensed HIV^TP^ particles with ultracentrifugation of the supernatants of COS-7 cells producing HIV_CA_^I2-V3^, immediately lysed the virions and subjected the virion lysates to WB, similarly large amounts of ladder-like degradation products were identified, confirming that the degradation products are also present within the virions (Fig. 1c). When HEK-293 cells were employed instead of COS-7 cells for transfection, similar ladder-like degradation products were observed in all the cell lysates producing HIV^TP^-containing the insert in CA (Fig. 1d). In order to determine whether other HIV-1-derived proteins were present in the degradation products, we subjected the cell lysates of COS-7 producing various HIV^TP^ including HIV_CA_^I2-V3^ to WB using an MA-specific monoclonal antibody (mAb). No MA-derived degradation products were identified in any of the cell lysates of HIV^TP^-producing COS-7 cells (Fig. S2a). When an Env-specific pAb was used, no additional Env-derived products were identified (Fig. S2b). These data confirmed that the observed degradation occurred in CA proteins, not in MA proteins or Env glycoproteins.

### Time-dependent progression of the CA degradation in HIV^TP^s and recombinant CA-containing 19-AA insert, and morphological profiles of HIV^TP^s-containing 19-AA insertion in the CA region

**Figure 2:**
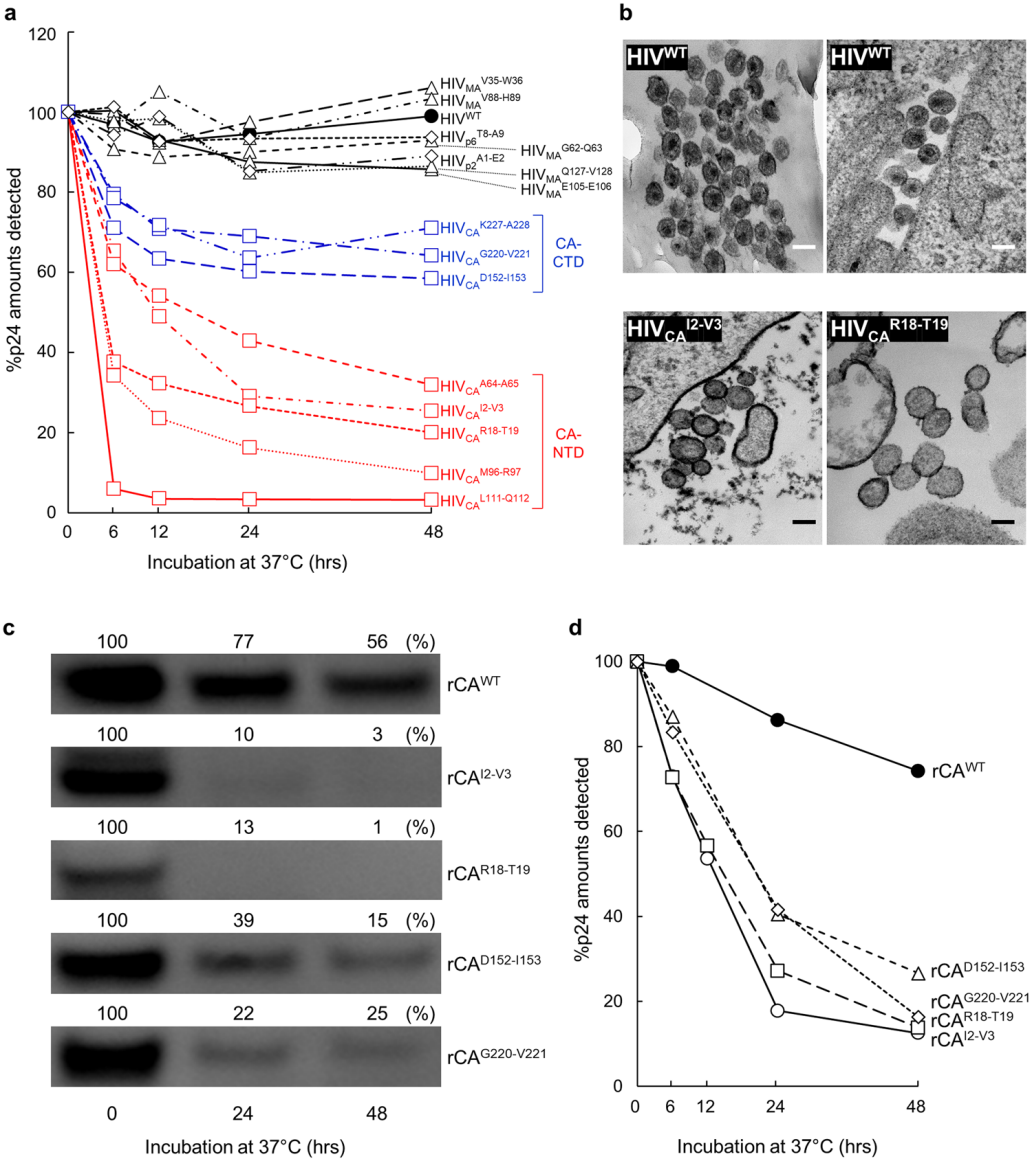
Time-dependent progression of the CA degradation in HIV^TP^s and recombinant CA-containing 19-AA insert. **(a)** Cell lysates of HIV^WT^- or various HIV^TP^-producing COS-7 cells were incubated at 37 °C for different periods of time, and the CA concentration of each sample was determined using ELISA. The sample at time 0 was stored at −80 °C immediately following the cell lysate preparation. “Percent p24 (CA)” was determined using equation described in the Methods section. Note that the lysates of COS-7 cells producing a panel of HIV-1-containing the 19-AAs in CA-NTD most rapidly lost CA, followed by the cells producing HIV-1 with the insert in CA-CTD. The data shown represent mean values derived from the results of two independent experiments. **(b)** Morphology of HIV_CA_^I2-V3^ and HIV_CA_^R18-T19^. HIV^WT^, HIV_CA_^I2-V3^ and HIV_CA_^R18-T19^ producing transfected COS-7 cells were subjected TEM study, as described in the Methods section. Note that HIV^WT^ virions had the typically-condensed mature core, while the cores within HIV_CA_^I2-V3^ and HIV_CA_^R18-T19^ are much less condensed or absent. The frequency of mature and aberrant cores are 75.7% (56/74) and 24.3% (18/74) for HIV^WT^ virions; 25.2% (27/107) and 74.8% (80/107) for HIV_CA_^I2-V3^ virions; 8.7% (2/23) and 91.3% (21/23) for HIV_CA_^R18-T19^ virions, respectively. Scale bars: 100 nm. **(c)** Various recombinant CAs such as wild-type HIV-1 CA (rCA^WT^) and the 19-AAs-containing CAs (rCA^I2-V3^, rCA^R18-T19^, rCA^D152-I153^, and rCA^G220-V221^) were expressed in COS-7 cells, the lysates of the cells incubated at 37 °C, subjected to WB using anti-p24 anti-serum, and the signal densities of rCAs quantified using the NIH Image J Program. Percent p24 (CA) was determined using equation described in the Methods section. The unincubated samples (0 hr) were normalized by their p24 concentrations (5 ng/well). 24 hrs and 48 hrs incubated samples were applied as same volumes as each unincubated (0 hr) sample. Results shown is representative of two independent experiments. **(d)** The same samples as in panel c were subjected to ELISA. Note that all the 19-AAs-containing CAs examined rapidly lost the immunogenicity as compared with rCA^WT^. Results shown is representative of two independent experiments. Full-length blots/gels are presented in Fig. S9.

We also examined if the observed CA degradation described above progressed when the lysates of COS-7 cells that produce various HIV^TP^ were incubated over different periods of time at 37 °C using ELISA that employed two CA-specific mAbs (Lumipulse *f*). Figure 2a shows the changes in the amounts of CA in each cell lysate. The %CA at each time point was calculated as the percentage relative to the amounts of detectable CA in unincubated cell lysates. CA degradation generally occurred by 6 hours of incubation and relatively slowly progressed by up to 24-hours afterwards (Fig. 2a). The reduction of the amounts of CA was the greatest in the lysates of HIV^TP^-CA^INS^NTD-producing COS-7 cells (range: 96.8~68.1% reduction at 48-hours) compared to HIV^TP^-CA^INS^CTD-producing cells (range: 41.5~28.9% at 48-hours) (Fig. 2a). Virtually no reduction in the amounts of CA was seen in the cell lysates of COS-7 producing HIV^WT^ or HIV^TP^s-containing the insert in MA, p2, or p6 proteins (Fig. 2a). These data are completely in line with our observation noted above (Fig. 1b,d) that the amounts of CA degradation products were greater in HIV^TP^-CA^INS^NTD-producing cells than in HIV^TP^-CA^INS^CTD-producing cells and that no CA degradation products were seen in the cell lysates of HIV^WT^ or HIV^TP^s-containing the insert in MA, p2, or p6 proteins. These data also strongly suggest that as CA degradation occurred, the two antigenic epitopes, located in the amino-terminus of CA, were lost, which the two CA-specific mAbs used in the Lumiplulse *f* system (https://soled.co/pdf-docs/fujirebio-lumipulse-f-service-manual) should otherwise recognize. When we examined whether the CA degradation progressed time-dependently using virion lysates from the supernatants of COS-7 cell cultures, the greatest reduction was seen in the virion lysates with HIV_CA_^I2-V3^ (HIV^TP^-CA^INS^NTD), followed by the lysates with HIV_CA_^D152-I153^ (HIV^TP^-CA^INS^CTD)(Fig. S3a). No reduction was seen in the virion lysates with HIV^WT^, HIV_MA_^Q127-V128^, or HIV_p2_^A1-E2^. When an MA-specific mAb was employed in WB, no degradation product’s signals were seen even after the lysates were incubated over the 40-hours (Fig. S2c).

Next, in order to determine if the CA degradation induced by the 19-exogenous-AA insertion had any effects on the morphology of virions, we conducted transmission electron microscopic (TEM) analysis using HIV^TP^-CA^INS^NTD-producing COS-7 cells. In the TEM images of HIV^WT^ produced by COS-7 cells, the typically-condensed mature core morphology was seen (Fig. 2b, upper panels). In contrast, in the TEM of HIV^TP^-CA^INS^NTD-producing COS-7 cells, most virions are rather greater in their sizes and the cores within the virions are much less condensed or absent, strongly suggesting that the CA in such virions significantly degraded and failed to be assembled (Fig. 2b, lower panels).

We further constructed plasmids to express recombinant HIV^WT^ CA (rCA^WT^) and recombinant CA-containing the 19-AA insert at different positions (two recombinant rCA^INS^NTD proteins: rCA^I2-V3^ and rCA^R18-T19^; two recombinant rCA^INS^CTD proteins: rCA^D152-I153^ and rCA^G220-V221^) and attempted to examine their structural integrity. As assessed with WB, rapid and drastic reduction in the signals of CA was seen within 24-hour incubation of the two rCA^INS^NTDs and moderate but yet drastic reduction was seen with the two rCA^INS^CTDs (Fig. 2c). The reduction levels became further greater by 48-hour incubation (Fig. 2c). The %signal-density reduction values in rCA^WT^, rCA^I2-V3^, rCA^R18-T19^, rCA^D152-I153^, and rCA^G220-V221^ were 23, 90, 87, 61, and 78% at 24-hours and 44, 97, 99, 85, and 75% at 48-hours, respectively, as determined using the NIH Image J Program. The ladder-like degradation products were also observed in unincubated COS-7 cell lysates expressing rCA^INS^NTDs and rCA^INS^CTDs (Fig. S3b). When the amounts of detectable CA were determined using ELISA, rapid reduction was seen within 24-hours with both rCA^INS^NTD and rCA^INS^CTD (Fig. 2d). However, the reduction of CA amounts was much less in rCA^WT^ (Fig. 2c,d; the actual amounts determined using the Lumipulse *f* are illustrated in Table S2).

### The CA degradation observed has no association with cellular/viral proteolytic mechanisms

**Figure 3:**
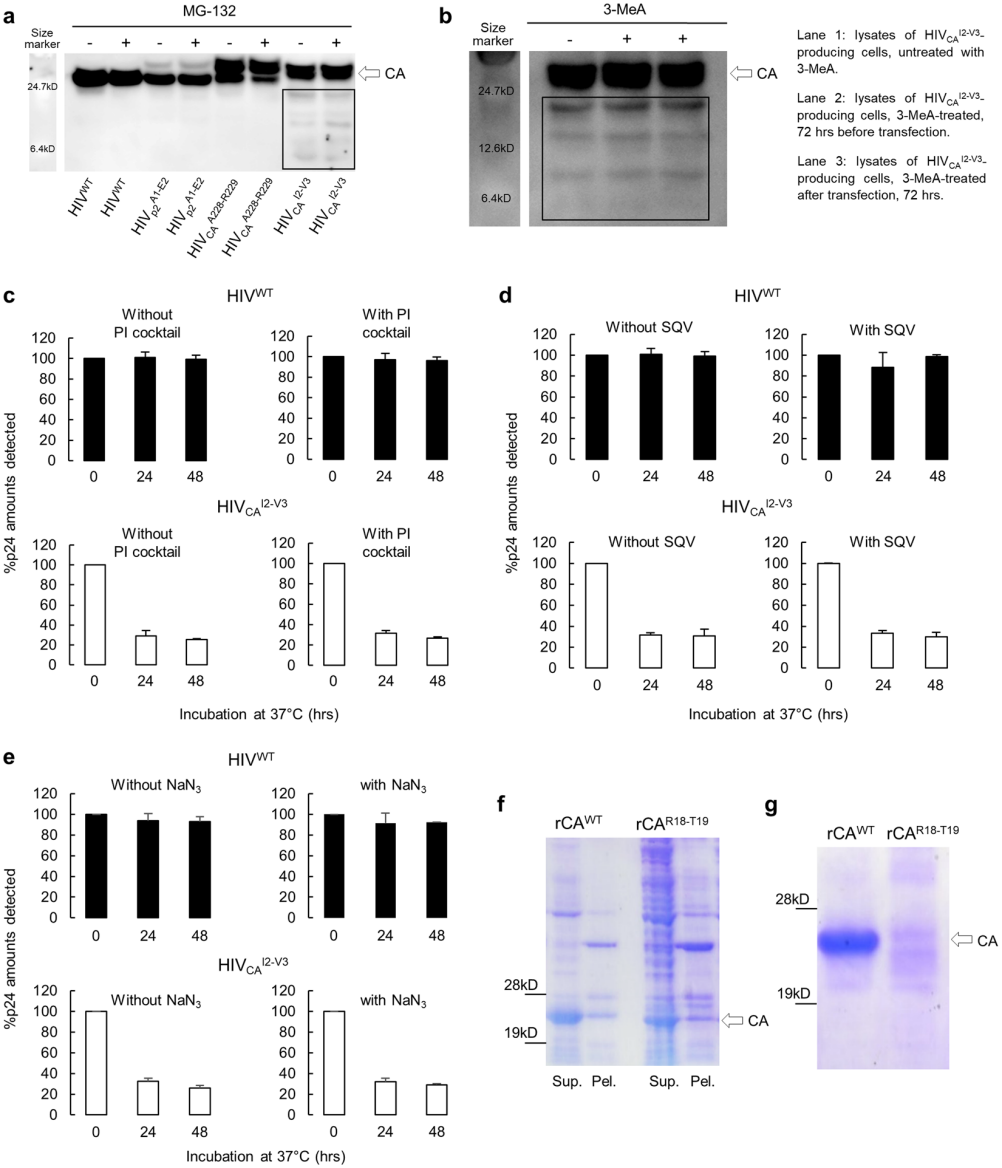
The CA degradation observed has no association with cellular/viral proteolytic mechanisms. **(a–d)** MG-132, an inhibitor of proteasome; 3-MeA, an autophagy inhibitor; a mixture of 7 potent cellular protease inhibitors; and SQV, a potent HIV-1-derived aspartyl protease failed to block CA degradation. **(a)** The lysates of COS-7 cells producing HIV^WT^ or HIV^TP^s were prepared 72 hrs after transfection and subjected to WB with anti-p24 polyclonal antibody. MG-132 was added to transfection medium with 20 μM concentration at 6 hrs before harvest. Results shown is representative of four independent experiments. **(b)** The lysates of HEK-293 cells producing HIV^WT^ or HIV^TP^_CA_^I2-V3^ were prepared 72 hrs after transfection and subjected to WB with anti-p24 polyclonal antibody. HEK-293 cells were incubated for 5 hrs with 5 mM of 3-MeA and were washed just before transfection (lane 2) or were incubated with 3-MeA for 72 hrs after transfection (lane 3). Results shown is representative of two independent experiments. All samples in **(a)** or **(b)** were normalized by their protein concentrations, 30 or 20 μg/well, respectively. **(c,d)** The lysates of COS-7 cells producing HIV^WT^ or HIV^TP^_CA_^I2-V3^ were prepared 72 hrs after transfection with lysis buffer-containing the PI cocktail or 1 μM of SQV. Data are represented as mean ± S.D. of two independent experiments. **(e)** The lysates of COS-7 cells producing HIV^WT^ or HIV_CA_^I2-V3^ were prepared with lysing buffer-containing 13.4 mM of sodium azide, incubated at 37 °C for different periods of time indicated and subjected to ELISA. Note that the presence of a high concentration of sodium azide did not block the CA degradation, suggesting that the degradation event is ATP-independent process. Data are represented as mean ± S.D. of two independent experiments. **(f)** rCA^WT^ and rCA^R18-T19^ produced by *E. coli* staining with Coomassie Brilliant Blue (CBB) are shown. rCA^WT^ and rCA^R18-T19^ were expressed in *E. coli* and each cultured cell preparation was lysed and ammonium-sulfate-precipitated. Subsequently, the lysed samples and ammonium-sulfate-precipitates were subjected to SDS-PAGE and CBB staining. **(g)** The ammonium-sulfate-precipitates of the rCA^WT^ and rCA^R18-T19^ samples described above were run through size-exclusion-chromatography and the fractions-containing rCA^WT^ and rCA^R18-T19^ were subjected to SDS-PAGE and CBB staining. Results shown is representative of two independent experiments. Full-length blots/gels are presented in Fig. S9.

We attempted to elucidate the mechanism of the CA degradation described above. Mammalian cells physiologically have multiple protein degradation pathways to regulate the levels of proteins. Such degradation pathways mainly include (i) the ubiquitin/proteasome^20^, (ii) autophagy^21^, and (iii) protease-associated proteolysis. Hence, we examined whether any of these degradation mechanism is responsible for the observed CA degradation. First, to examine whether the ubiquitin/proteasome system was in operation, we employed MG-132, a proteasome inhibitor, which caused the accumulation of ubiquitinated proteins at the concentration of 20 µM (Fig. S4a). However, the same concentration of MG-132 failed to block the CA degradation (Fig. 3a). 3-MeA, an autophagy inhibitor, blocked the formation of autophagosome (Fig. S4b); however, this inhibitor also failed to block the CA degradation (Fig. 3b). In order to ask whether cellular proteases were responsible for the CA degradation, we used a mixture of 7 cellular proteases inhibitors that fairly well blocked trypsin digestion of BSA (Fig. S4c), while the same mixture completely failed to block the CA degradation (Fig. 3c). It was also possible that HIV-1-derived aspartyl protease might have caused the CA degradation since precursors of CA proteins are digested by retrovirally-expressed protease. Thus, we used HIV-1 protease-inhibitor, saquinavir (SQV, 1 µM), which efficiently blocked HIV-1 protease activity (Fig. S4d). However, SQV also failed to block the CA degradation (Fig. 3d).

A number of proteins in both prokaryotic and eukaryotic cells are subject to energy-dependent proteolysis. Since sodium azide is known to inhibit the production of ATP through inhibition of oxidative phosphorylation^22^, we examined whether the CA degradation observed is an ATP-dependent process. When we added >10 mM sodium azide that is known to cause ATP-depletion^22^ to the lysates of HIV_CA_^I2-V3^-producing cells, no inhibition of the CA degradation was observed (Fig. 3e), strongly suggesting that the CA degradation observed is not an energy-dependent process.

We also examined whether the degradation also occurred when rCA^WT^ and rCA^R18-T19^ were expressed in *E. coli* and size-exclusion-chromatographically purified so that no human cellular components or HIV-1 viral components were involved in the degradation process. The lysates of *E. coli* were centrifuged to afford CA-containing supernatants, from which proteins were precipitated with 20% ammonium sulfate. In both the supernatants and precipitates obtained, p24 fractions-containing rCA^WT^ or rCA^R18-T19^ were clearly identified as examined with SDS-PAGE and Coomassie-blue staining (Fig. 3f). When both rCA^WT^ and rCA^R18-T19^ were fractionated with size-exclusion-chromatography and both samples were further examined with SDS-PAGE and Coomassie blue, rCA^WT^ signals were readily identified; however, rCA^R18-T19^ signals were almost absent while ladder-like signals were identified (Fig. 3g) that were seen when COS-7 and HEK-293 cells producing HIV_CA_^R18-T19^ were examined (Fig. 1b,d). These data strongly suggest that degradation of rCA^R18-T19^ proceeded during the fractionation and SDS-PAGE procedures, while rCA^WT^ remained intact.

Taken together, these results strongly suggest that no cellular/viral proteolytic mechanisms are involved in the CA degradation observed and that the degradation is not energy-dependent. Thus, we assumed that the CA degradation observed occurs most likely due to compromised structural integrity caused by the 19-AA insertion into CA.

### The degradation of mutant CAs requires the cleavage of the C-terminus of CA and originates from the CA-CTD

**Figure 4:**
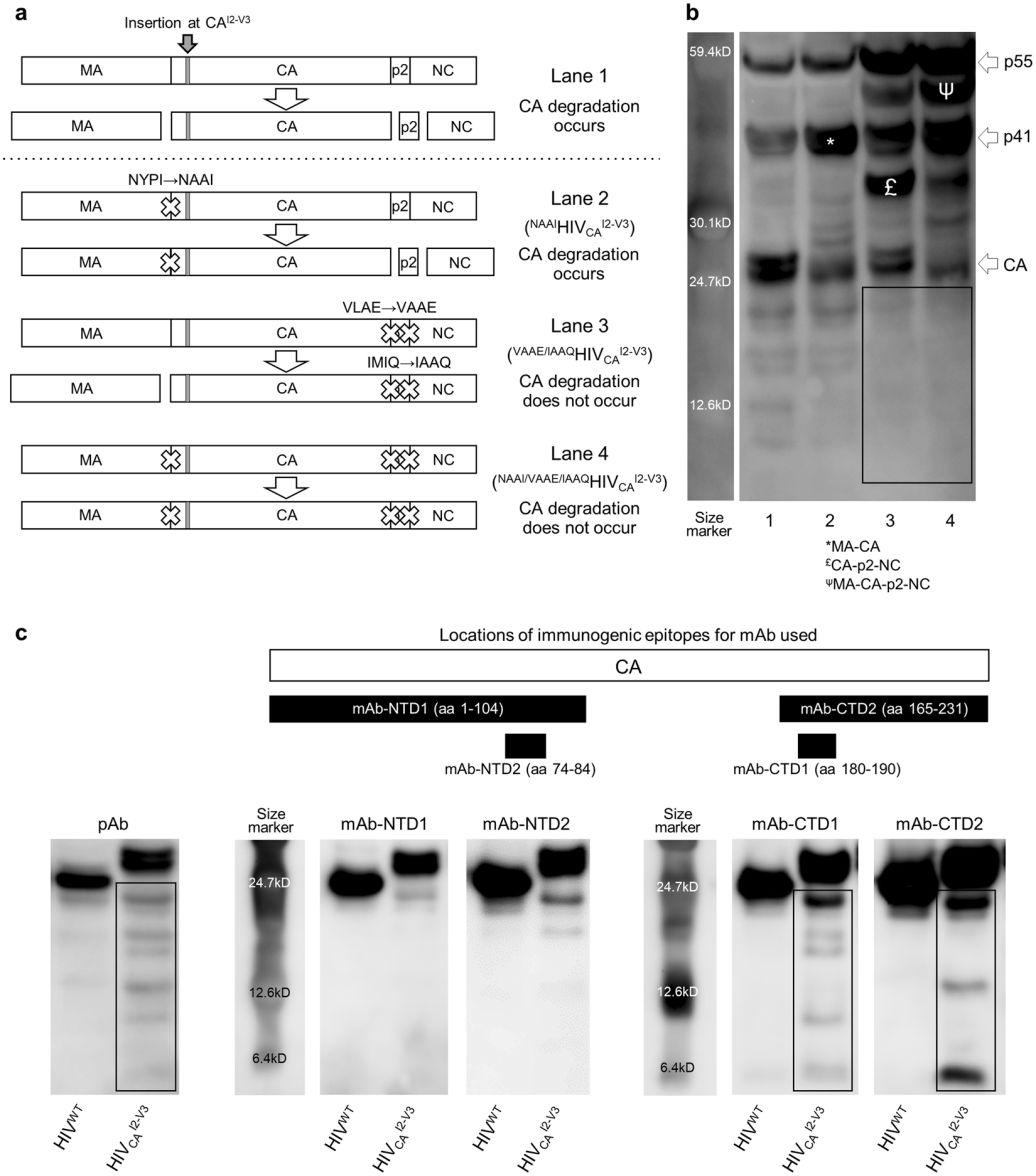
The degradation of mutant CAs requires the CA’s C-terminal cleavage and originates from the CA-CTD. **(a)** A genetic map of the three following HIV_CA_^I2-V3^-based constructs lacking the cleavage(s) of Gag polyproteins generated: (i) a construct, in which the MA-CA cleavage of HIV_CA_^I2-V3^ was blocked by changing the cleavage site “NYPI” to “NAAI” (thus referred to ^NAAI^HIV_CA_^I2-V3^), (ii) a construct, in which the CA-p2 and p2-NC cleavages were blocked by changing the two cleavage sites “VLAE” and “IMIQ” to “VAAE” and “IAAQ”, respectively (^VAAE/IAAQ^HIV_CA_^I2-V3^), and (iii) a construct, in which all the three cleavages were blocked (^NAAI/VAAE/IAAQ^HIV_CA_^I2-V3^). **(b)** Reduced degradation in ^VAAE/IAAQ^HIV_CA_^I2-V3^ and ^NAAI/VAAE/IAAQ^HIV_CA_^I2-V3^. The lysates of COS-7 cells producing HIV_CA_^I2-V3^ (Lane 1), ^NAAI^HIV_CA_^I2-V3^ (Lane 2), ^VAAE/IAAQ^HIV_CA_^I2-V3^ (Lane 3), or ^NAAI/VAAE/IAAQ^HIV_CA_^I2-V3^ (Lane 4) were subjected to WB using anti-p24 anti-serum. CA degradation was clearly seen in Lanes 1 and 2, while the degradation levels in Lanes 3 and 4 were drastically reduced, indicating that the blockade of cleavages in the CA-p2-NC cleavage sites resulted in reduction of the CA degradation. **(c)** The CA^I2-V3^ degradation products are derived from the CTD of CA^I2-V3^. The lysates of HEK-293 cells producing HIV_CA_^I2-V3^ were subjected to WB using two monoclonal antibodies specific to the CA-NTD (mAb-NTD1 and mAb-NTD2), and two monoclonal antibodies specific to the CA-CTD (mAb-CTD1 and mAb-CTD2). The locations of each immunogen recognized are shown in the top of Panel **c**. Note that the ladder-like signals were identified only in the lysates examined with the two CA-CTD-specific mAbs. All samples in **(b,c)** were normalized by their protein concentrations (20 μg/well). Results shown is representative of three to five independent experiments for each mAb. Full-length blots/gels are presented in Fig. S9.

We then investigated how the CA degradation is initiated and proceeds. CA, as a critically important viral protein, is under strong selective pressure to regulate its production, maturation, integral structure, and functions. In the replication cycle of HIV-1, Gag polyprotein undergoes cleavage and maturation to form the functional CA. Thus, we examined the relationships of the cleavage sites that flank the CA precursor in the polyprotein. We generated three HIV_CA_^I2-V3^-based constructs that lack the cleavage(s) of Gag polyprotein (Fig. 4a): (i) a construct, in which the MA-CA cleavage of HIV_CA_^I2-V3^ was blocked by changing the cleavage site from “NYPI” to “NAAI” (thus referred to ^NAAI^HIV_CA_^I2-V3^), (ii) a construct, in which the CA-p2 and p2-NC cleavages were blocked by changing the two cleavage sites from “VLAE” to “VAAE” and “IMIQ” to “IAAQ” (^VAAE/IAAQ^HIV_CA_^I2-V3^), and (iii) a construct, in which all the three cleavages were blocked (^NAAI/VAAE/IAAQ^HIV_CA_^I2-V3^)(Fig. 4a). In COS-7 cells producing ^NAAI^HIV_CA_^I2-V3^, the accumulation of the MA-CA precursor protein was seen as expected, while in COS-7 cells producing ^VAAE/IAAQ^HIV_CA_^I2-V3^ and ^NAAI/VAAE/IAAQ^HIV_CA_^I2-V3^, the accumulation of the CA-p2-NC precursor protein and MA-CA-p2-NC precursor protein were seen, respectively (Fig. 4b). Of note, the CA^I2-V3^ degradation products were identified only in COS-7 cells producing HIV_CA_^I2-V3^ and ^NAAI^HIV_CA_^I2-V3^, but virtually absent in those producing ^VAAE/IAAQ^HIV_CA_^I2-V3^ and ^NAAI/VAAE/IAAQ^HIV_CA_^I2-V3^ (Fig. 4b, boxed). These data showed that the blockade of the CA^I2-V3^ cleavage at the C-terminal end resulted in the absence or notable decrease of CA^I2-V3^ degradation.

We further attempted to determine the constituents of the CA^I2-V3^ degradation products in terms of NTD or CTD products using polyclonal anti-CA anti-serum, two mAbs specific to the CA-NTD [mAb-NTD1 and mAb-NTD2 recognizing immunogens at the N-terminus, aa1–104 and aa74–84, respectively], and two mAbs specific to the CA-CTD [mAb-CTD1 and mAb-CTD2 recognizing immunogens at the C-terminus, aa180–190 and aa165–231, respectively]. When the lysates of HEK-293 cells producing HIV_CA_^I2-V3^ were subjected to WB using anti-CA pAb, the ladder-like signals were seen in HIV_CA_^I2-V3^-producing cells but not in HIV^WT^-producing cells (the very left panel in Fig. 4c). However, as examined with mAbs, the ladder-like signals were identified only in the lysates examined with the two CA-CTD-specific mAbs (Fig. 4c). Of note, the signals seen with the pAb are those with mAb-CTD1 added with those with mAb-CTD2 (Fig. 4c). These findings were corroborated when the lysates of HEK-293 cells producing five other HIV-1 constructs (HIV_CA_^R18-T19^, HIV_CA_^A64-A65^, HIV_CA_^M96-R97^, HIV_CA_^L111-Q112^, and HIV_CA_^D152-I153^) were subjected to WB using mAb-NTD1 or mAb-CTD1. Significant amounts of CA degradation products were seen as examined with pAb and mAb-CTD1, while virtually no significant degradation products were identified as examined with mAb-NTD1 (Fig. S5).

Taken together with the data above that the CA^I2-V3^ cleavage blockade at the C-terminus end resulted in the absence or notable decrease of CA^I2-V3^ degradation (Fig. 4b), the results strongly suggest that CA degradation observed is initiated at the CTD and proceed in a time-dependent manner.

### Degradation-prone HIV^TP^-CA^INS^ variants are replication-incompetent

**Figure 5:**
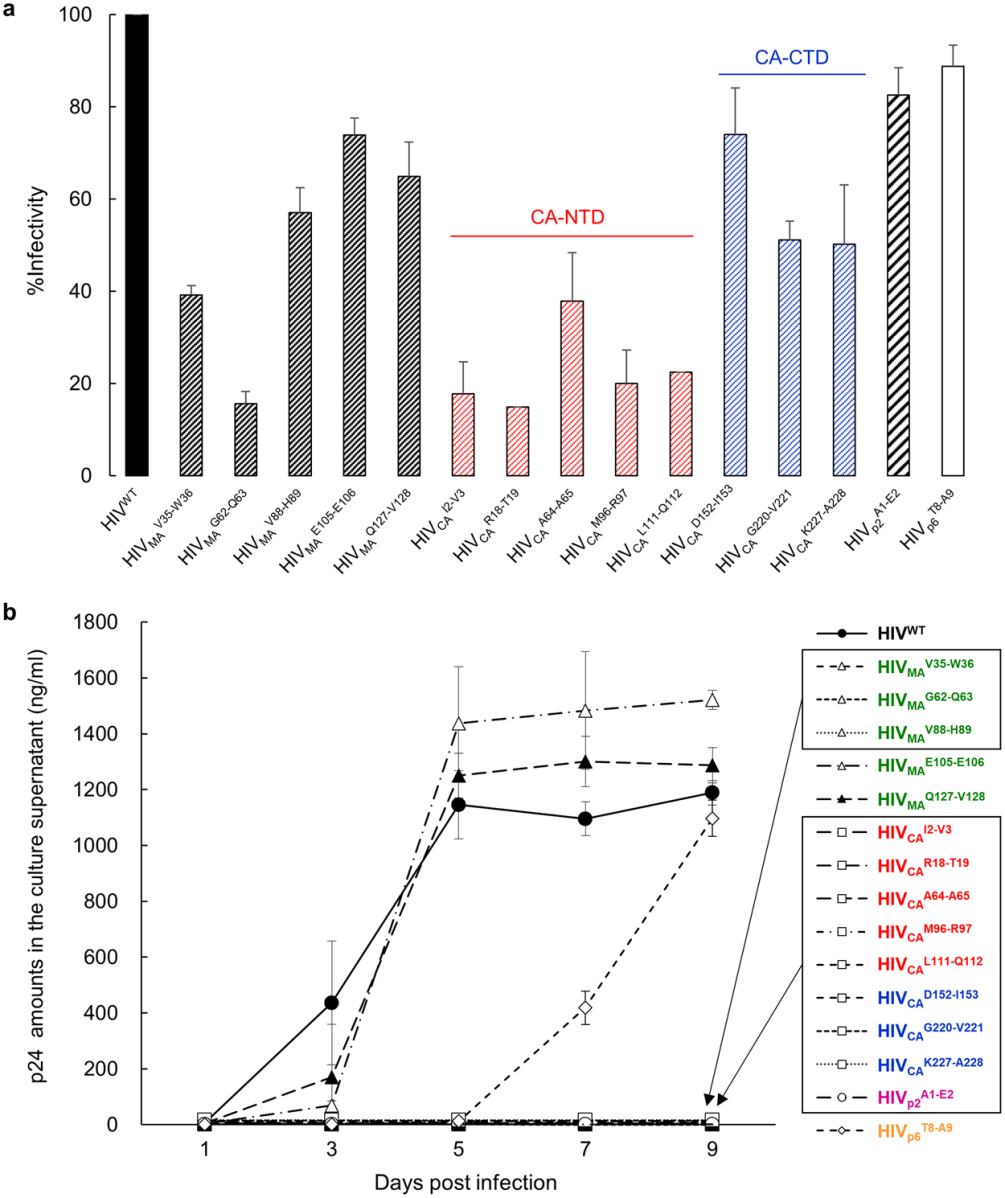
Degradation-prone HIV^TP^-CA^INS^ variants are replication-incompetent. **(a)** The infectivity of each of HIV^TP^-CA^INS^NTDs (shown in red) and HIV^TP^-CA^INS^CTDs (in blue) was determined with the single-round infectivity assay using U373-Magi^CD4+CXCR4+^ cells. Note that the infectivity of HIV^TP^-CA^INS^s was substantially reduced, while that of three HIV^TP^-MA^INS^s (HIV_MA_^V35-W36^, HIV_MA_^G62-Q63^, and HIV_MA_^V88-H89^) were also substantially reduced. Data are represented as mean ± S.D. (n = 3). **(b)** Loss of replication capacity of HIV^TP^-CA^INS^NTDs (in red) and HIV^TP^-CA^INS^CTDs (in blue). Replication kinetics of HIV^TP^-CA^INS^s was determined with the amounts of p24 production in cultures of MT-4 cells exposed to each HIV^TP^. The boxes denote that all HIV^TP^-CA^INS^s examined completely failed to replicate in MT-4 cells, while the three HIV^TP^-MA^INS^ (in green) described above and HIV_p2_^A1-E2^ (in purple) also failed to replicate. HIV_p6_^T8-A9^ (in orange) showed slower replication in MT-4 cells compared to HIV^WT^. Data are represented as mean ± S.D. (n = 2).

We next determined the single-round infectivity of HIV^TP^ virions harvested from the culture supernatants of transfected COS-7 cells with the MAGI assay using U373-Magi^CD4+CXCR4+^ cells. All five HIV^TP^-CA^INS^NTDs virions examined (HIV_CA_^I2-V3^, HIV_CA_^R18-T19^, HIV_CA_^A64-A65^, HIV_CA_^M96-R97^, and HIV_CA_^L111-Q112^) that had high-level CA degradation (Figs 1b,d and 2a) showed drastically reduced infectivity compared to HIV^WT^ virions (Table 1 and Fig. 5a). The three HIV^TP^-CA^INS^CTDs virions examined (HIV_CA_^D152-I153^, HIV_CA_^G220-V221^, and HIV_CA_^K227-A228^) that had moderate-level CA degradation (Figs 1 and 2) had rather moderately reduced infectivity compared to the five HIV^TP^-CA^INS^NTDs virions examined. When we determined the replication kinetics of HIV^TP^-CA^INS^NTDs and HIV^TP^-CA^INS^CTDs virions using the MT-4 cell-based replication assay, both the virion groups examined had completely lost their replication capacity (Table 1 and Fig. 5b). Interestingly, HIV^TP^s-containing the AA insertion in the NTD of MA (HIV_MA_^V35-W36^, HIV_MA_^G62-Q63^, and HIV_MA_^V88-H89^), none of which showed CA degradation (Figs 1b and 2a) but had moderately to drastically reduced infectivity (Fig. 5a), showed no replication capacity (Fig. 5b). In addition, one HIV^TP^-containing the AA insertion in the p2 protein, which showed no CA degradation (Figs 1b and 2a) or no drastic infectivity reduction, had also completely lost its replication capacity (Table 1 and Fig. 5).

**Table 1:** Summary of CA Degradation Levels, Single-Round Infectivity, and Replication Capacity of Wild-Type HIV-1 and Various 19-AA-Containing HIV-1 Variants.

WT or variant	%CA^a^	Infectivity^b^	Replication^c^
HIV^WT^	96.3	100.0	+
HIV_MA_^V35-W36^	106.1	39.2	−
HIV_MA_^G62-Q63^	92.9	15.6	−
HIV_MA_^V88-H89^	103.3	48.5	−
HIV_MA_^E105-E106^	85.7	73.9	+
HIV_MA_^Q127-V128^	86.5	64.9	+
HIV_CA_^I2-V3^	25.4	17.7	−
HIV_CA_^R18-T19^	20.0	14.8	−
HIV_CA_^A64-A65^	31.9	37.8	−
HIV_CA_^M96-R97^	9.9	20.0	−
HIV_CA_^L111-Q112^	3.2	22.4	−
HIV_CA_^D152-I153^	58.5	74.0	−
HIV_CA_^G220-V221^	64.2	51.1	−
HIV_CA_^K227-A228^	71.1	50.2	−
HIV_p2_^A1-E2^	89.0	84.0	−
HIV_p6_^T8-A9^	93.7	82.1	+

^a^%Immunogenicity of CA protein 48 h incubation at 37 °C (Fig. 2a). ^b^Percentage of X-gal positive cells in wells exposed to HIV^WT^ or each HIV-1 variant as assessed in Magi assay (Single-round infectivity; Fig. 5a). ^c^Results of replication of HIV^WT^ or each variant as assessed in MT-4 cells (Fig. 5b). (+) indicates that the virus replicated virtually comparably to HIV^WT^.

### Co-expression of pHIV^WT^ and pHIV^TP^-CA^INS^NTD produces highly compromised or replication-incompetent HIV-1

**Figure 6:**
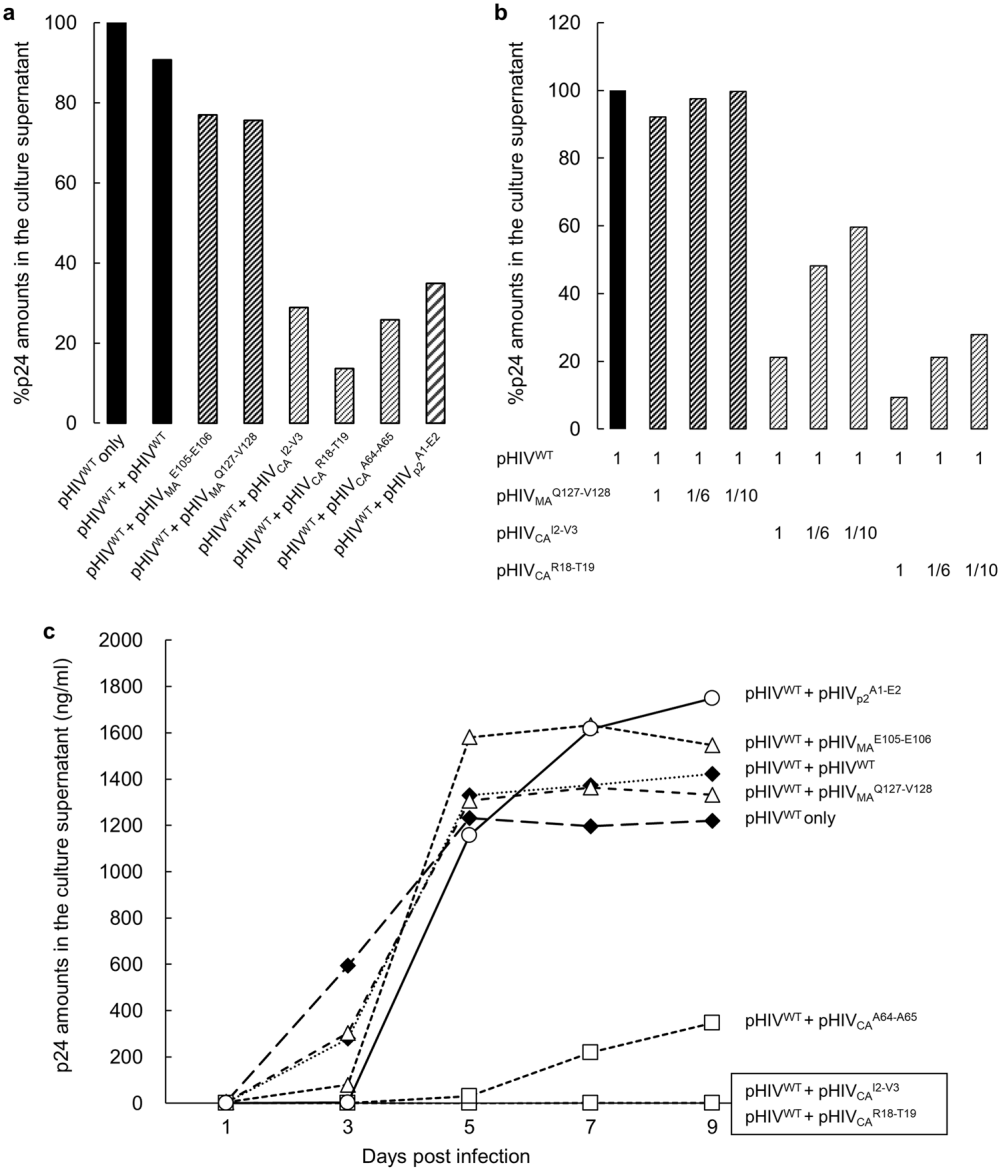
Co-expression of pHIV^WT^ and pHIV^TP^-CA^INS^NTD produces highly compromised or replication-incompetent HIV-1. **(a)** HEK-293 cells were transfected with pHIV^WT^ alone, pHIV^WT^ plus pHIV^WT^, pHIV_MA_^E105-E106^, pHIV_MA_^Q127-V128^, pHIV_CA_^I2-V3^, pHIV_CA_^R18-T19^, pHIV_CA_^A64-A65^ or pHIV_p2_^A1-E2^, cultured for 72 hrs, and the p24 amounts of each culture supernatant were determined. Note that when co-transfected with pHIV^WT^ and either of 3 pHIV^TP^-CA^INS^NTD (pHIV_CA_^I2-V3^, pHIV_CA_^R18-T19^, pHIV_CA_^A64-A65^) and pHIV_p2_^A1-E2^, p24 production was drastically reduced as compared when transfected with pHIV^WT^ and pHIV^WT^ plus pHIV^WT^. **(b)** HEK-293 cells were transfected with pHIV^WT^ alone, co-transfected with 1:1, 1:1/6, and 1:1/10 ratios of pHIV^WT^ and pHIV_p17_^Q127-V128^, pHIV_CA_^I2-V3^, or pHIV_CA_^R18-T19^, cultured for 72 hrs, and the p24 amounts of each culture supernatant were determined. Results shown is representative of three independent experiments. **(c)** The supernatants harvested from each of the cultures described in Panel a were subjected to 9-day HIV-1 replication assays using MT-4 cells as target cells, after all the supernatants were normalized to contain an equal amount of p24. Note that the supernatants from transfection with pHIV^WT^ alone or pHIV^WT^ plus pHIV^WT^, pHIV_p2_^A1-E2^, pHIV_MA_^E105-E106^, or pHIV_MA_^Q127-V128^ produced large amounts of p24 over the 9-day culture period, while those from transfection with pHIV^WT^ plus either of the three pHIV^TP^-CA^INS^NTD (pHIV_CA_^I2-V3^, pHIV_CA_^R18-T19^, pHIV_CA_^A64-A65^) produced no or least p24 protein. All p24 values are single-point determinations.

We then attempted to examine the impact of CA^INS^NTD production onto the replication competence of HIV^WT^ by co-transfection of HEK-293 cells with pHIV^WT^ and pHIV^TP^-CA^INS^NTD. pHIV^TP^-CA^INS^NTD has all the HIV^WT^ genetic components except the CA-encoding gene carrying the 19-AAs in the NTD. When cells were transfected with pHIV^WT^ only, a large amount of p24 (516 ng/ml) was produced, while co-transfected with pHIV^WT^ and either of 3 pHIV^TP^-CA^INS^NTD (pHIV_CA_^I2-V3^, pHIV_CA_^R18-T19^, and pHIV_CA_^A64-A65^), p24 production was drastically reduced by 71.1, 86.4, and 74.2%, respectively, strongly suggesting that the co-expression of rCA^WT^ with rCA^I2-V3^, rCA^R18-T19^, and rCA^A64-A65^ caused drastically enhanced degradation of the presumably chimeric rCA molecules comprised of rCA^WT^ and rCA^INS^NTD (Fig. 6a). In order to corroborate and extend these results, we performed co-transfection experiments using different ratios of pHIV^WT^ and pHIV^TP^ amounts. When HEK-293 cells were co-transfected with 1:1, 1:1/6, and 1:1/10 ratios of pHIV^WT^ and pHIV_MA_^Q127-V128^ amounts, no drastic reduction of p24 amounts produced was seen (Fig. 6b). In contrast, co-transfection of 1:1/6, and 1:1/10 ratios of pHIV^WT^ and pHIV_CA_^I2-V3^ amounts caused as much as 51.8 and 40.4% reduction in the amounts of detectable p24. Co-transfection of 1:1/6, and 1:1/10 ratios of pHIV^WT^ and pHIV_CA_^R18-T19^ amounts also caused 78.8 and 72.1% reduction in the amounts of detectable p24 (Fig. 6b).

The supernatants harvested from each of the transfectant cultures of HEK-293 cells (Fig. 6a) were subsequently subjected to HIV-1 replication assays. After all the supernatants were normalized to contain an equal amount of p24 (10 ng/ml), serving as a source of infectious virions. MT-4 cells were exposed to each supernatant, washed, and cultured over 9 days. The supernatants from HEK-293 cells transfected with pHIV^WT^ alone produced large amounts of p24 proteins (~1,200 ng/ml) by the end of the 9-days-culture period. Of note, MT-4 cells exposed to the supernatants of HEK-293 cells transfected with a two-fold greater amount of pHIV^WT^ (pHIV^WT^ plus pHIV^WT^ as a control for other co-transfections) produced comparable amounts of p24 (~1,200 ng/ml) as compared to the amounts with only pHIV^WT^ transfection (Fig. 6c). However, MT-4 cells exposed to the supernatants from HEK-293 cells, which were co-transfected with pHIV^WT^ plus either of the three pHIV^TP^-CA^INS^NTD, produced no or the least p24 proteins over the 9-days-culture period, indicating that those supernatants contained virions with substantially compromised replication capacity or replication-incompetent virions. These data also strongly suggest that the presence of the three rCA^INS^NTD caused the failure of the formation of functional CA, resulting in replication inhibition of the otherwise replication-competent HIV^WT^. HEK-293 cells co-transfected with pHIV^WT^ and either of two pHIV^TP^-MA^INS^ (pHIV_MA_^E105-E106^ and pHIV_MA_^Q127-V128^) produced moderately reduced p24 in the culture (Fig. 6a); however, MT-4 cells exposed to those culture supernatants produced comparable p24 amounts compared to the supernatants from transfection with pHIV^WT^ alone and with pHIV^WT^ plus pHIV^WT^ (Fig. 6c). MT-4 cells exposed to the supernatants from HEK-293 cells co-transfected with pHIV^WT^ plus pHIV_p2_^A1-E2^ also produced p24 proteins comparably to the supernatants from transfection with pHIV^WT^ alone (Fig. 6c).

### Modelling study of the mechanism of the CA degradation

**Figure 7:**
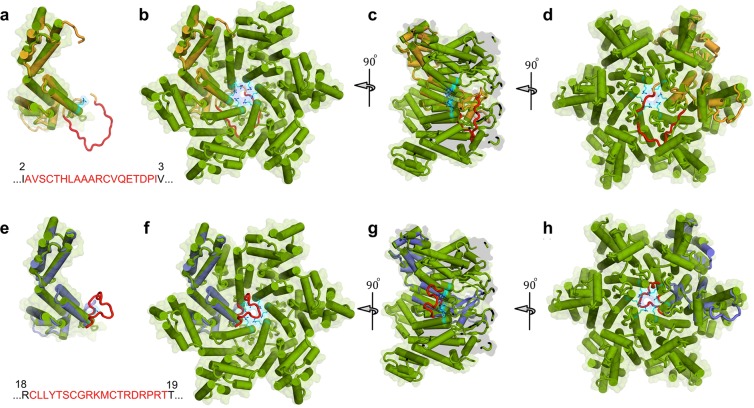
Modeling study of the mechanism of the CA degradation. The 19-AA inserts are indicated by red color for both CA^I2-V3^ and CA^R18-T19^. On the upper panel, the model of CA^I2-V3^ in orange are superimposed into the monomer **(a)** and the hexamer **(b–d)** forms of the CA^WT^ structure (PDB ID: 5HGL). Similarly, lower panel dedicated to the structural comparison of the CA^R18-T19^ model colored in purple with the CA^WT^ monomer **(e)** as well as the hexamer form **(f–h)** structures. Hexamers are shown from top views **(b**,**f)**, 90°-rotated side views **(c**,**g)**, and 180°-rotated views **(d**,**h)**. Note that the presence of 19-AA inserts let to formation of extended loops, which wrap around the CA pore composed of 6 arginine residues (shown in cyan). It is assumed that the extended loops are likely to push each monomer apart and effectively block the formation of physiologic hexamer complexes.

In an attempt to delineate the possible mechanism of the CA degradation, we first generated 19-AA insertion models and examined how the insertions could pose structural and functional impacts to each CA monomer that is to form hexamers (Figs 7, S6, and Movie S1). In the case of CA^I2-V3^, the insertion I^2^-V^3^ is located at the very N-terminal region (Fig. 7a–d) and is highly likely to effect the beta-hairpin loop that has crucial function by stabilizing hexamer complex. The view of the hexamer comprised of six CA^I2-V3^ monomers (Fig. S6d) strongly suggests that the insert should disrupt the structure and integrity of the pore, whose function is reportedly required for nucleotide entry to fuel RT activity inside the CA shell^23^. In the case of CA^R18-T19^, the insert R18-T19 is also located in the proximity of the N-terminal region of CA monomer and is thought to do the same as does the insertion I^2^-V^3^. In fact, both of the two insertions (I^2^-V^3^ and R^18^-T^19^) resulted in a total loss of the replication capacity of HIV-1 carrying those inserts (HIV_CA_^I2-V3^ and HIV_CA_^R18-T19^), most likely resulting from significantly disturbed structural and functional integrity of CA. In the cases of CA^A64-A65^, CA^M96-R97^, and CA^L111-Q112^, where the 19-AA insertion occurred in the proximity of H4, H5, and H6, which, unlike the cases of CA^I2-V3^ and CA^R18-T19^, are rather distant from the N-terminal region. As seen in the Fig. S6a, the insert is located relatively distant form the center of the hexamer and seems to cause less drastic effects on the structure of the hexamer and the pore. Although the levels of CA degradation in and the incompetent replication capacity of HIV_CA_^A64-A65^, HIV_CA_^M96-R97^, and HIV_CA_^L111-Q112^ were similar to those in HIV_CA_^I2-V3^ and HIV_CA_^R18-T19^, when COS-7 cells was co-transfected with pHIV^WT^ and pHIV_CA_^A64-A65^, the replication of the otherwise totally replication-incompetent pHIV_CA_^A64-A65^ was moderately rescued (Fig. 6c), in contrast to the co-transfection of COS-7 cells with pHIV^WT^ plus pHIV_CA_^I2-V3^ and pHIV^WT^ plus pHIV_CA_^R18-T19^, which resulted in no identifiable rescue-effect by pHIV^WT^ was seen (Fig. 6c). These data should suggest that the insert, which occurred rather distant from the N-terminal region, posed relatively moderate detrimental impact to the structure and function of CA hexamers. When the insertion occurred in the C-terminal region of CA (HIV_CA_^D152-I153^, HIV_CA_^G220-V221^, and HIV_CA_^K227-A228^), the insert apparently does not interfere with essential intramolecular interactions and the flexible 19-AAs sequence could adopt different conformation without blocking hexamer formation (Fig. S6a) and the insert probably does not pose severe crippling effects on the structure or functions of the hexamers. In fact, the levels of CA degradation in the three infectious clones are only moderate (Figs 1b,d and 2a) and their infectivity was also only moderate (Fig. 5a). The structural examination of the locations of the insert and the phenotypic features of each clone with the insert strongly suggest that the impacts of the insert are drastically affected by the location of the insert, as were seen in the cases of inserts Tamiya *et al*. reported causing significant CA degradation (^19^; Fig. S1a). However, it should also be noted that, while the levels of CA degradation, the rescue-effects by co-transfection with pHIV^WT^, and the infectivity assessed in the single-round HIV-1 replication assay were moderate or not-too-debilitating, all the 19-AAs-containing clones were virtually equally replication-incompetent (Fig. 5b). In this regard, it is rather understandable that for the occurrence of successive HIV-1 replication, robust structural and functional integrity of CA is required^24,25^. Also, we considered that the 19-AA insertion at the CA-NTD induced conformational alternations that extended into the distant C-terminal region of CA resulting in an unstable CA-CTD and the induction of degradation initiation at the CTD as shown in Fig. 4, suggesting that the 19-AA insertion at the CA-NTD causes gross conformational changes, which extended to the CA-CTD, resulting in structural fragility in both CA domains. Taken together, the present results suggest that the CA-p2 and p2-NC cleavage sites should be intact or the structural integrity of the CA-p2-NC domains should also be kept intact for the infectivity and replication capacity of HIV-1 and that if either (or both) of these molecular events is affected, HIV-1 CA degradation is initiated in the CTD and the degradation of virtually entire CA ensues.

## Discussion

The class of HIV-1 PIs represents a major component of the currently available cART of HIV-1 infection and AIDS, which has been shown to potently suppress the replication of HIV-1 and significantly extend the life expectancy of HIV-1-infected individuals^26^. It has been well reported that in the course of cART including PIs, HIV-1 acquires AA substitutions in the PR-encoding gene and avoids the binding of PIs to PR, thus developing resistance to PIs. The introduction of such AA substitutions into PR, however, often results in compromised proteolytic activity of PR, causing impaired replication competence of the virus^11,27–29^. In compensation of the reduced catalytic activity of mutant PRs, HIV-1 is known to add AA substitutions/insertions in the cleavage sites of the Gag proteins, substrates for PR, to restore the otherwise compromised enzymatic cleavage activity of mutated PRs, enabling multi-PI-resistant HIV-1 variants to keep or restore replication-competence^19,30–32^. Moreover, certain AA substitutions in non-cleavage sites within the Gag proteins have been shown to contribute to the development of higher levels of HIV-1 resistance to PIs^33,34^.

In the present study, we first found that drug-resistant HIV-1 carrying multiple AA insertions in Gag undergoes aberrant CA degradation (^19^; Fig. S1a). To elucidate the mechanism of the aberrant degradation and to determine the role of AA insertions into CA in the infectivity and replication capacity of HIV-1, we generated a series of recombinant HIV-1 clones-containing a 19-AA insertion randomly introduced into the Gag region using the Tn5-based transposon system (HIV^TP^). Although 19-AA insertion is relatively long and can be conformationally bulkier and rather more complex than the inserts identified in HIV-1 Gag in the context of HIV-1’s acquisition of resistance to PIs^19^, but we chose the 19-AA insertion in the present study since it is technically efficient and less labor-intensive than the site-directed mutagenesis to generate various replication-competent HIV-1 variants with AA insertions in CA and elucidate the effects of such AA insertions on the behavior of the Gag polyproteins in drug-resistant HIV-1 variants. The 19-AA insertion *per se* probably does not have structural fragility or instability, in view that the same AA insertion in MA underwent no undue degradation (Fig. S2a,c).

If one carefully takes a look at our previous paper by Tamiya *et al*.^19^, no significant consecutive amino acid substitutions or repeats were observed or described except for one or two sporadic substitutions in the CA-encoding region. These data should strongly suggest that if such significant amino acid substitutions (*i.e*., 3 to 6 or longer consecutive amino acids insertions) occur in the CA-encoding region, such mutant HIV-1 viruses would lose their replicable fitness, presumably due to the CA degradation we have seen in the current study, and are not seen in the clinical setting. In this regard, the integrity of CA and its formation is more susceptible to the impact of 3 or longer consecutive amino-acid insertions in CA rather than in matrix or p6. We would assume that the magnitude of CA degradation and the loss of viral replicability should be associated with both “the size and location of the inserts”. The longer the insert, the greater the magnitude of degradation and compromised replication. At this time, we have not directly asked whether “smaller AA sequences” would promote the degradation and compromised replication, which we are afraid goes beyond the scope of the current study. However, Tamiya *et al*. generated a recombinant clone HIV-1_NL4–3_ containing the “SQVN” insert, located 8 amino-acid-upstream from the I2-V3 insert of HIV_CA_^I2-V3^ (HIV^SQVN^), which proved that HIV^SQVN^ showed significant capsid degradation as well as compromised replicability^19^. Thus, such previous data by Tamiya *et al*. and our current data should strongly suggest that the significant CA degradation observed is caused by the inserts (3 or longer consecutive amino acids) and is associated with the loss of replicability.

In Tamiya’s report, the magnitude of the compromised replicative fitness of recombinant clones engineered to have 3 to 6 AA insertions was not that great (downed by ~20-fold compared to HIV-1_NL4–3_)^19^ compared to the recombinant clones engineered to have 19-AA inserts in CA presented in the current study. This difference should be, as we mentioned above, due to that the integrity of CA and its formation is more susceptible to the impact of AA insertions in CA rather than in matrix or p6 documented in Tamiya’s report^19^. In this regard, we have newly conducted an experiment, in which a recombinant clone engineered to contain two inserts, AQQA and PTAPPA inserts that did not cause “severe” reduction in viral replication in Tamiya’s report, designated HIV_MA_^AQQA^_p6_^PTAPPA^. As we expected, this HIV_MA_^AQQA^_p6_^PTAPPA^ had significant CA degradation and “severe” reduction in replicability (Fig. S8).

The CA degradation identified also progressed in a time-dependent manner when incubated at 37 °C (Fig. 2). Moreover, the CA degradation was shown to begin at the CA-CTD as examined using multiple different CA-specific mAbs, which recognized different antigenic epitopes of CA (Fig. 4c). Also, the data strongly suggest that proteasome, autophagy, or cellular/viral proteases are not responsible for the CA degradation (Fig. 3a–d). In addition, the CA degradation does not seem to be an energy-dependent process (Fig. 3e). Moreover, CA degradation also occurred when rCA^R18-T19^ was expressed in *E. coli* (Fig. 3f,g). Altogether, it is assumed that the CA degradation is caused by the structural instability and/or compromised structural integrity (Fig. 3).

The mature CA mainly exists as a hexamer and the CA-NTD appears to play an important role in its formation in view of both geographical and functional perspectives. In this regard, Jacques and his colleagues have shown that each CA hexamer has a size-selective pore bound by a ring of six Arg-18 residues and a ‘molecular iris’ is formed by the amino-terminal β-hairpin^23^. In this respect, a study of >70,000 protein structures and complexes for unusual formation of arginine residues by Neves *et al*.^35^ has shown that arginines form various clusters that are exposed to interact with and potentially be controlled or switched by nucleic acids, charged metabolites, membrane lipids, and side chains of other proteins. Also, Mallery *et al*. reported that these pores bind the cellular polyanion IP6, transforming viral stability and allowing newly synthesized DNA to accumulate inside the CA^36^. These data, together with the data on CA hexamers^23^ strongly suggest that the control of the stability endowed by arginine clusters may play an important role in protein-protein oligomerization, molecular recognition, DNA accumulation, and ligand binding. Interestingly, the CA degradation and the loss of infectivity and replication of HIV-1 were most profound in the present study when 19-AA insertions were added to CA-NTD (residues 1–145) involving the arginine clusters, while the loss of infectivity was less extensive when the insertions were added to CA-CTD (residues 151–231), distant from the CA-NTD. The loss of replication capacity of HIV-1s that contained the 19-AA insertions in the CA-NTD was relatively more drastic than their infectivity was probably since the replication capacity represents the results of multiple cycles of HIV-1 replication and would show more significant compromised results.

It is of note that all HIV^TP^-CA^INS^NTDs and HIV^TP^-CA^INS^CTDs totally failed to replicate in MT-4 cells, although HIV^WT^ and three other rHIV^TP^s (HIV_MA_^E105-E106^, HIV_MA_^Q127-V128^, and HIV_p6_^T8-A9^) vigorously replicated (Fig. 5b). The replication failure in all the recombinant HIV-1 carrying TP at the CA-NTD and CA-CTD should stem from the disturbed structural integrity of CA. In this regard, when the morphological abnormality is drastic as examined with TEM, no replication capacity is seen as easily expected; but with much less or even apparently normal morphology of the virions, the virus virtually completely loses replication capacity^37^. These observations suggest that the induction of even nominal CA abnormality could possibly completely abrogate the replication capacity of HIV-1. Indeed, when we assessed the morphology of two HIV^TP^-CA^INS^NTDs (HIV_CA_^I2-V3^ and HIV_CA_^R18-T19^), which totally failed to replicate in MT-4 cell culture (Fig. 5b), the cores of those virions were much less condensed or absent as compare to normal virions (Fig. 2b). These drastic morphological abnormalities are fairly in line with the failure of their replication loss. In the relevant context, the co-expression of HIV^TP^-CA^INS^NTD with HIV^WT^ notably impaired the replication capacity of HIV^WT^ (Fig. 6c), probably supports the “assembly defect” of the chimeric virions with physiologically robust CA plus structurally compromised CA due to the insert would diminish or nullyfy the replication capacity. Indeed, CA^INS^NTD expressed from co-transfected mutant HIV^TP^s probably incorporated into CA multimers within HIV^WT^ virions, thereby disrupting the formation of normal CA shells and resulting in viral replication failure (Fig. 6c).

Also, it is of note that CA-CTDs contribute to hexamer-hexamer interactions during the formation of a conical shaped mature CA shell in HIV-1 virions^38,39^. Thus, we hypothesize that the 19-AA insertions in the CA-NTD directly interfere with CA hexamer formation and that the degradation initiation induced at CA-CTD additionally prevents CA-CTD-mediated hexamer-hexamer interactions. Our TEM studies also support this notion, revealing that normal CA cores are not present in HIV^TP^-CA^INS^NTD virions (Fig. 2b). It is possible that the two distinct structural effects mentioned above (the presence of insertion in the NTD and following CA degradation initiated from CTD) induce this drastic morphological abnormality in the HIV^TP^-CA^INS^ virions.

With regard to our failure to determine the exact mechanism of the CA degradation we have observed other than the possible spontaneous degradation due to the disintegrity of the CA structure, the best study would be to determine the X-ray structure of 19-AA insertion-containing CA. However, it should be probably impractical to express and crystalize 19-AA insertion-containing CA due to its highly degradation-prone property. Indeed, CA has the most unique biological feature: a certain level of structural stability of CA conical shell is needed for the protection of viral RNA copies and other enzymes such as reverse transcriptase, while at the same time the CA shell should be “brittle” and “unstable” to disintegrate upon “uncoating” around the intracellular entry of HIV-1, eventually allowing the transport of the viral pre-integration complex into the cytoplasm, then to the nucleus. In fact, even “when, where, and how CA uncoating occurs” still remains hypothetic. It is not known as to (i) whether “uncoating” takes place immediately after the HIV-1 fusion with the cellular membrane; (ii) whether a partial uncoating occurs within the cytoplasm; or (iii) whether uncoating occurs at the nuclear pore. Whether viral/cellular factors are involved in the CA uncoating process is also presently unclear^40^. Nevertheless, physiological CA degradation is obviously initiated upon/through the uncoating process. We believe that the 19-AA insertion in CA induces conformational/structural disintegrity, significantly enhancing the CA degradation. In fact, as illustrated in Fig. 1d, WB results of HIV^WT^ cell lysates show multiple signals although with less intensity compared to the degradation products of 19-AA insertion-containing CA, such as HIV_CA_^I2-V3^. It should be noted that the weak signals in the HIV^WT^ cell lysates are of the same molecular weights as in the degradation products of 19-AA insertion-containing CA, strongly suggesting that the 19-AA insertion potentiated the otherwise slow and modest physiological CA degradation. Indeed, in recombinant CA^WT^ expressed in *E. coli*, similarly weak degradation signals at the same mobilities as seen in recombinant CA^R18-T19^, were also identified (Fig. 3g).

The reason why cells co-transfected with pHIV^WT^ plus pHIV_p2_^A1-E2^ produced a notably reduced amount of p24 protein is not readily understood, although the supernatants had contained robustly replication-competent virions (Fig. 6a,c). However, it is noted that the insert had occurred between alanine-1 and glutamic acid-2 of p2, that is located just next to the cleavage site CA^L231^-p2^A1^. Thus, it is possible that the structural conformation of the cleavage site CA^L231^-p2^A1^ was drastically altered and the cleavage efficiency was compromised. The notable restoration of the infectivity and replication capacity seen when co-expression of pHIV^WT^ plus pHIV_p2_^A1-E2^ was made well explain since physiological p2 produced with pHIV^WT^ rescued the otherwise compromised and non-replicative HIV^WT^ (Fig. 6c).

The WB results showed that recombinant CA-containing the 19-AAs in both NTD and CTD underwent drastic degradation in time-dependent manner, although CA degradation in HIV^TP^-CA^INS^NTD and rCA^INS^NTD underwent substantially greater levels of degradation than in HIV^TP^-CA^INS^CTD and rCA^INS^CTD (Figs 1b,d and 2a,c). Of note, when the quantities of CA were determined as p24 antigen levels using a quantitative immunoassay employing two CA-specific mAbs, the CA signals were rapidly lost over the 48-hour incubation of various HIV^TP^-CA^INS^NTD (Fig. 2a). Although the CA signals were also lost when HIV^TP^-CA^INS^CTD were similarly placed at 37 °C, the intensities of the loss were moderate compared to those of HIV^TP^-CA^INS^NTD. It is noteworthy that the degradation of rCA^WT^ was not drastic even after 48-hour incubation as assessed with WB using a pAb against CA, although in the cases of rCA^I2-V3^ and rCA^R18-T19^, both of which carry the insert in the CA-NTD, CA degradation rapidly progressed within 24-hours and virtually no drastic signals were detected in 48-hours. When the amounts of CA in two rCA^INS^NTDs (rCA^I2-V3^ and rCA^R18-T19^) and two rCA^INS^CTD (rCA^D152-I153^ and rCA^G220-V221^) were quantified using the automated quantitative immunoassay, the signals were also progressively lost over the incubation. When using another manufacturer’s ELISA kit to corroborate these results, similar degradation profiles were observed (Fig. S7). These data strongly suggest that CA degradation was so extensive when incubated over 24 to 48 hours at 37 °C that most of the antigenic epitopes on CA that would be detected by the anti-serum had been nearly entirely destroyed. It is worth noting that when we attempted to determine the AA-sequences of each of the CA degradation product ladders using the pieces of nitrocellulose membrane-containing the ladders with the Edman degradation method^41^; however, we failed to obtain the AA-sequences.

HIV^TP^s carrying the insert at the NTD of MA (HIV_MA_^V35-W36^, HIV_MA_^G62-Q63^, and HIV_MA_^V88-H89^) had reduced infectivity and replication capacity (Table 1 and Fig. 5); however, none of them had notable protein degradation (Figs 1b and 2a). In this regard, the positions V35 and W36 of HIV_MA_^V35-W36^ are located in the basic domain of MA, which plays an important role in Env incorporation over the assembly stage. The positions G62/Q63 and V88/H89 are located in the proximity of or within the “virus assembly domain” and “Gag-targeting domain”^39^, respectively, both of which are essential for HIV’s acquisition of infectivity and replicative ability. Thus, the AA insertion in the NTD of MA presumably disrupts these functional domains.

Most importantly, in the present study, when we examined the impact of CA^INS^NTD production onto the replication competence of HIV^WT^ conducting co-transfection of cells with pHIV^WT^ and pHIV^TP^-CA^INS^NTD, the resultant probably “chimeric” virions showed highly compromised replication capacity or replication-incompetence (Fig. 6). The p24 found in the supernatants of the co-transfected COS-7 cells shown in Fig. 6a should represent p24 from replicative HIV-1 (chimeric HIV containing CA^WT^ plus mutated MA or mutated p2) and p24 from non-replicative HIV-1 (chimeric HIV containing CA^WT^ plus mutated CA). Figure 6c shows that the chimeric HIV containing CA^WT^ plus mutated MA or mutated p2 were capable of replicating; while the chimeric HIV containing CA^WT^ plus mutated CA failed to replicate in the case of co-transfection with pHIV_CA_^I2-V3^ and pHIV_CA_^R18-T19^. The data shown in Fig. 6c strongly suggest that the mutant CA such as CA^I2-V3^ and CA^R18-T19^, if integrated into the capsid-shell formed with CA^WT^, induces degradation of the whole CA. In other words, these data strongly suggest that the incorporation of degradation-prone (unstable or fragile) CA^INS^NTD into the otherwise functional CA results in drastic disruption of the formation of normal CA shells, leading to the loss of replication competence in such resultant “chimeric” virions (Fig. 6c). It is possible that if certain mutated CA-encoding genetic materials could be introduced to express such degradation-prone CA species within the cells productively infected with HIV^WT^ or even drug-resistant HIV, the resultant “chimeric virions” would lose their replication capacity, causing “intracellular killing of infectious virions” and eventual cellular apoptosis without production of infectious virions, a potentially new modality of reduction or elimination of the HIV-1-infected cells.

In conclusions, the present observations of CA degradation may allow us to understand how CA decomposes and possibly the dynamics and mechanism of the formation of CA hexamers and decomposition/degradation process upon the initial phases of HIV-1’s cellular entry. The present observation may also open a new avenue for discovery of new antiretroviral modalities to compromise the orderly CA degradation and the uncoating process, or new anti-HIV-1 agents inducing abnormal CA degradation.

## Methods

### Cells

MT-4 cells were grown in RPMI 1640-based culture medium supplemented with 10% fetal calf serum (FCS; PAA Laboratories GmbH, Linz, Austria) plus 50 U of penicillin and 100 μg of kanamycin per ml. COS-7 and HEK-293 cells (DS pharma biomedical, Osaka) were grown in DMEM-based culture medium supplemented with 10% FCS plus 50 U of penicillin and 100 μg of kanamycin per ml. U373-MAGI^CD4+CXCR4+^ cells were obtained through the AIDS Reagent Program, NIAID, NIH, were maintained in DMEM with 10% FCS, 0.2 mg/ml G418, 0.1 mg/ml hygromycin B, and 1.0 µg/ml puromycin.

### Inhibitors of cellular/viral protein degradation pathways/enzymes

To determine whether cellular/viral protein degradation pathway/enzyme have any effects on the CA degradation observed, we employed the following inhibitors: (i) a proteasome inhibitor, MG-132 (Sigma-Aldrich Japan, Tokyo, 20 µM); (ii) a cocktail of seven mammalian protease inhibitors (PIs; Sigma): AEBSF (final concentration, 1 mM), aprotinin (0.8 μM), bestatin (40 μM), E-64 (10 μM), leupeptin (100 μM), pepstatin (15 μM), and phosphoramidon (10 μM); (iii) an autophagy inhibitor, 3-methyladenine (3-MeA, Sigma, 5 mM); and (iv) an HIV-1 PI, saquinavir (SQV, 1 μM), which was kindly provided by Roche Products Ltd. (Welwyn Garden City, United Kingdom).

### Generation of recombinant HIV-1 clones

We generated a series of HIV-1_NL4-3_-based recombinant clones containing a 19-AA insert randomly introduced into Gag proteins using the Tn5-based transposon system (EZ-Tn5™ In-Frame Linker Insertion Kit, Epicentre, Madison, WI) according to the manufacture’s protocol. In brief, a plasmid (TA cloning kit^TM^ with pCR^TM^ 2.1 vector, Thermo Fisher Scientific, Waltham, MA) containing the *Bss*HII*-Sma*I fragment of the wild-type HIV-1_HXB2_ (nucleotides 712 to 2591, encoding Gag and PR) was exposed to EZ-Tn5™ transposon, which contains the kanamycin-resistance-associated gene, and transposase, followed by *E. coli* transformation and kanamycin selection. The resulting colonies with 19 AA-containing *Bss*HII*-Sma*I fragment was picked up, kanamycin-resistance-associated gene was removed by *Not*I restriction enzyme, and HIV-1_NL4-3_ constructs with the 19 AA-containing *Bss*HII*-Sma*I fragment were generated using pHIV-1_NLSma_, which had been generated to have a *Sma*I site by changing two nucleotides (2590 and 2593) of pHIV-1_NL4-3_, and expanded. Thus obtained 19 AA-containing Gag-encoding gene was subjected to DNA sequencing and the amino acid sequences and locations of the 19 AA within Gag were determined through deducing the nucleic acid sequences. Insert-containing CA-encoding plasmids were generated as follows. pCMV-Myc (Takara Bio, Kusatsu, Shiga) was digested with ApaI/XhoI to remove Myc-epitope and linearize the vector. CA fragment of each recombinant HIV-1 clone was amplified with PCR, and each of thus-generated fragments was introduced into the linearized pCMV-Myc using In-fusion enzyme (Clontech Laboratories, Takara bio, Kusatsu, Shiga).

### Western blot analysis

To analyze whether HIV-1 polyproteins in each of molecular HIV-1 clones generated were cleaved by HIV-1 PR, Western blot (WB) analysis with the lysates of HIV-1-producing cells and cell-free virions was conducted. Briefly, at 72 hours after transfection using Lipofectamine LTX (Invitrogen, Thermo Fisher Scientific, Waltham, MA) with plasmid preparations, COS-7 or HEK-293 cells were washed with PBS and lysed in M-per solution (Thermo Fisher Scientific, Waltham, MA), and the cell lysates were subjected to WB. Culture supernatants containing virions were collected 72 hours after transfection, filtered through Steriflip Millipore Express PLUS membranes (0.22 μm pore-size, Millipore, Bedford, MA), and centrifuged at 20,000 *g* for 24 hours to pellet virions, were then lysed in M-per solution. In the WB assay, samples were normalized based on the protein concentration and subjected to electrophoresis on sodium dodecyl sulfate 4–12% polyacrylamide gel, followed by electroblotting onto nitrocellulose membranes.

The HIV-1 Gag proteins and Env glycoprotein were visualized with SuperSignal WestPico (Thermo Fisher Scientific, Waltham, MA) and Can Get Signal (Toyobo, Kitaku, Osaka) by using anti-HIV-1 p24 (CA) polyclonal antibody (pAb; Advanced Biotechnologies, Eldersburg, MD, Cat# 13-203-000, RRID:AB_1929205), 4 different anti-HIV-1 CA monoclonal antibodies (mAbs; United States biological, Salem, MA, Cat# H6005-08A, -08B, -08D, and -08F), anti-HIV-1 p17 (MA) mAb (Advanced Biotechnologies, Cat# 13-103-100, RRID:AB_1929216), and anti-HIV-1 gp120 pAb (D7324; Aalto Bio Reagents, Dublin). We also used following polyclonal antibodies (pAbs); anti-ubiquitin pAb (Calbiochem, San Diego, CA Cat# 662099, San Diego, CA), anti-LC3 pAb (Novus Biologicals, Littleton, CO, Cat# NB100-2220, RRID:AB_10003146), anti-BSA pAb (BioVision, Milpitas, CA, Cat# 5998-100, RRID:AB_2225924).

### Determination of time-dependent CA degradation

The cell/virion lysates were prepared 72 hours after transfection and incubated at 37 °C for different periods of time, and CA concentrations of each cell/virion lysates at optional time points were measured using a fully-automated chemiluminescent enzyme immunoassay system (Lumipulse *f*: Fujirebio Inc., Tokyo). Graphs were generated based on the percentage of the CA concentration in each lysate sample incubated at 37 °C for different time periods, compared to the CA concentration of the control samples, which had been stored at −80 °C immediately after preparation and served as 100%. Percent p24 (CA) represents the percentage determined using the following equation: 100 × (CA conc. of cell lysates at each incubation time)/(CA conc. of the time 0 lysates)(%).

### Determination of single-round infectivity and replication capacity of each recombinant infectious HIV-1 clone containing an insert in CA

To determine the single-round infectivity of each recombinant infectious clone containing an insert in CA, we conducted the Magi assay using U373-MAGI^CD4+CXCR4+^ cells^42^. The U373-MAGI^CD4+CXCR4+^ cells (6 × 10^4^ cells/500 µL/well) were cultured in DMEM containing 10% FCS in 24-well plates for 24 hours, exposed to each infectious clone (10 ng/ml of p24-equivalent/300 µL/well) for 2 hours, replenished with 1 mL fresh culture medium, and further cultured for another 48 hours. Following removal of the supernatants, the cells were fixed with 1% formaldehyde and 0.2% glutaraldehyde in PBS for 5 minutes, the fixing solution was removed, and the cells were washed with PBS and visualized with staining solution (950 μl PBS, 20 μl 0.2 M potassium ferrocyanide, 20 μl 0.2 M potassium ferricyanide, 1.0 μl 2.0 M Mg_2_Cl, and 10 μl 40 mg/ml X-gal stock) over 2 hours. Finally, the stained cells were washed twice with PBS and the number of blue-stained infected cells was determined under microscopy.

To determine the replication capacity of each recombinant infectious clones, replication kinetics assay employing fresh MT-4 cells was performed as previously described^43,44^. In brief, COS-7 cells were transfected with pHIV^WT^ or pHIV^TP^s and incubated for 3 days, then concentrations of p24 in culture supernatants were measured and stored at −80 °C until use. MT-4 cells (1 × 10^5^) were exposed to the each HIV^WT^ or HIV^TP^ preparation containing 10 ng/ml of p24 in 6-well culture plates for 5 hours, and those MT-4 cells were washed and cultured with fresh culture medium. The amounts of p24 in supernatants were determined every two days for up to 9 days.

### Transmission electron microscopy (TEM) analysis

COS-7 cells were transfected with pHIV^WT^, pHIV_CA_^I2-V3^, or pHIV_CA_^R18-T19^, centrifuged, fixed in phosphate-buffered 2% glutaraldehyde, post-fixed in 2% osmium tetra-oxide in ice, dehydrated in ethanol, and embedded in the epoxy resin. Ultrathin sections of the samples were stained with uranyl acetate for 10 min and with lead-staining solution for 5 minutes, and submitted to TEM (JEM-2000EX and JEM-1200, JEOL, Peabody, MA).

### Expression and purification of recombinant CA in *E.coli*

For the generation of recombinant CA expression vectors, the entire HIV-1 CA-encoding gene from pHIV_NL4-3_ or pHIV_CA_^R18-T19^ was introduced to pET30a (Merck KGaA, Darmstadt, Germany) using the In-Fusion method. The CA-encoding gene-containing pET30a was transformed into Rosetta^TM^ (DE3) pLysS Competent Cells (Novagen, Merck Millipore, Kenilworth, NJ) grown in LB medium supplemented with kanamycin and chloramphenicol to an OD_600_ of 0.5 at 37 °C, and the expression of rCA was induced with 1.0 mM isopropyl β-D-1-thiogalactopyranoside (IPTG) for 3–4 hours at 37 °C. The cells were sonicated in lysis buffer (5 mM 2MetOH, 150 mM NaCl, 50 mM Tris-HCl, pH 7.4), centrifuged for 15 min at 3,500 rpm, and the obtained supernatants were added with 20% of (NH_4_)_2_SO_4_, and the precipitates were collected with centrifugation for 15 min at 3,500 rpm. Thus obtained precipitates were resuspended in lysis buffer, centrfuged for 20 min at 15,000 rpm at 4 °C, and the supernatants were passed through 0.45 μm filter and loaded onto a GE Hi Load 16/600 Superdex (GE Healthcare, Chicago, IL) attached to a AKTA prime plus (GE Healthcare). rCA-containing fractions were pooled and concentrated using Amicon Ultra 10 K device (Merck Millipore, Kenilworth, NJ) in the lysis buffer. The concentration of protein was determined using BCA Protein Assay Reagent Kit (Thermo Fisher Scientific, Waltham, MA).

### Computational modeling of AA insertions into CA

Computational models of AA insertions at various sites of CA were generated using the Structure Prediction Server (PS)^2^ version 3.0^45^ using the complete sequence of the insertion along with the flanking sequences from the wild-type CA. The models generated were downloaded from the server and were analyzed using PyMol version 1.7.6.6 (Schrodinger, LLC). The flanking sequence of the obtained model was then superposed onto the corresponding matching sequence of the wild-type CA (PDB ID: 5HGL).

## Supplementary information

Supplementary materials

Movie S1

## Data Availability

All data generated or analyzed during this study are included in this published article and its Supplementary Information Files.
