# Supplementary material for: Amino-acid inserts of HIV-1 capsid (CA) induce CA degradation and abrogate viral infectivity: Insights for the dynamics and mechanisms of HIV-1 CA decomposition
Source: Sci Rep. 2019 Jul 8;9:9806. doi: 10.1038/s41598-019-46082-2 (PMC6614453; doi:10.1038/s41598-019-46082-2)

Supplemental materials for

# Amino-acid inserts of HIV-1 capsid (CA) induce CA degradation and abrogate viral infectivity: Insights for the dynamics and mechanisms of HIV-1 CA decomposition.

Masayuki Amano[1,2,3], Haydar Bulut[2], Sadahiro Tamiya[1,2], Tomofumi Nakamura[1], Yasuhiro Koh[1], and Hiroaki Mitsuya[1,2,3,#]

[1]Department of Hematology, Rheumatology, and Infectious Diseases, Kumamoto University School of Medicine, Kumamoto, Japan

[2]Experimental Retrovirology Section, HIV and AIDS Malignancy Branch, Center for Cancer Research, National Cancer Institute, National Institutes of Health, Bethesda, MD, USA

[3]Department of Refractory Viral Infection, National Center for Global Health and Medicine Research Institute, Tokyo, Japan

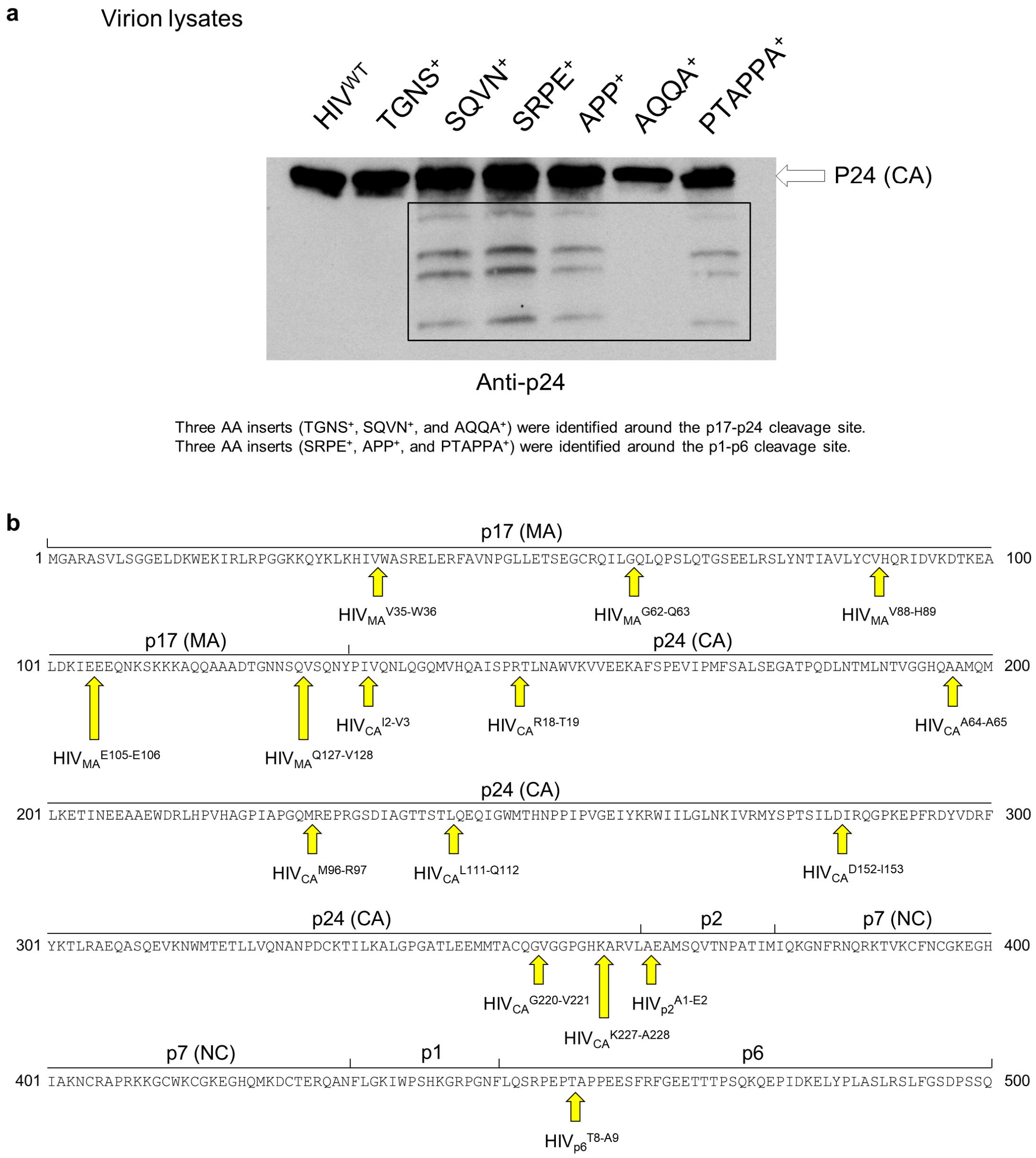


**Fig. S1. CA Degradation Observed in HIV-1 Variants with the AA Insertion in Gag Region, and Locations of Each 19-AA Insertion in Gag of HIV$^{TP}$ Clones Generated by Using the Tn5-based Transposon System.**

**(a)** CA degradation in HIV-1 carrying 3 to 6 AA insertions in Gag. Virion lysates obtained from COS-7 cells producing HIV-1$_{NL4-3}$ (HIV$^{WT}$) or HIV-1 carrying each insert were subjected to WB with CA (p24)-specific polyclonal anti-serum. The

virion lysates were prepared by centrifugation of the supernatants 48 h after transfection. The positions corresponding to the sizes of p24 indicated by arrows. Note that CA degradates are seen in HIV-1 containing 3 to 6 AA insertions: SQVN (in p17), SRPE (p6), APP (p6), and PTAPPA (p6) in addition to the known PI-resistance-associated multiple AA substitutions within the protease [19]. **(b)** Locations of each 19-AA insertion in Gag of $HIV^{TP}$ clones generated by using the Tn5-based transposon system are shown. For example, $HIV_{MA}^{V35\text{-}W36}$ and $HIV_{CA}^{I2\text{-}V3}$ contain the 19-AA insertion between V35 and W36 in MA, and I2 and V3 in CA, respectively. The AA sequence of $HIV^{WT}$ Gag is illustrated as a reference.

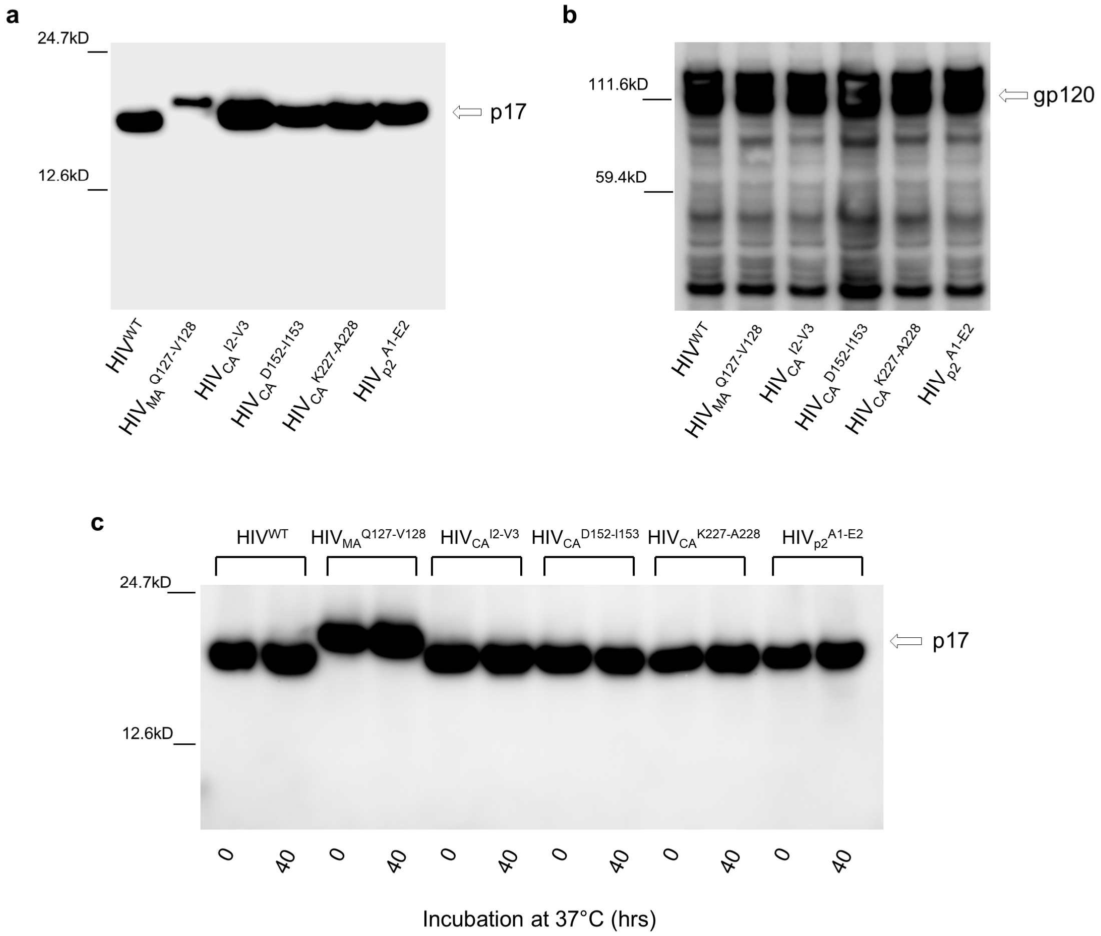


**Fig. S2. The Degradates Identified with Anti-p24 Antibody are not Observed in WB with Anti-p17 (MA) or Anti-gp120 (env) Antibodies.**

**(a** and **b)** Cell lysates of $HIV^{WT}$-, $HIV_{MA}^{Q127\text{-}V128}$-, $HIV_{CA}^{I2\text{-}V3}$-, $HIV_{CA}^{D152\text{-}I153}$-, $HIV_{CA}^{K227\text{-}A228}$-, $HIV_{p2}^{A1\text{-}E2}$-producing COS-7 cells following transfection and 72-hour culture, were subjected to WB assays using MA-specific monoclonal and Env (gp-120)-specific polyclonal antibodies. Note that no MA- or Env-derived degradates were identified. All samples were normalized by their protein concentrations (20 μg/well). **(c)** The COS-7 cell lysates described in Panel **(a)** was frozen at -80°C immediately after being harvested or incubated at 37°C for 40 hours, and subjected to WB assay with an MA-specific monoclonal antibody. Note that no degradate signals

other than MA-specific signals were seen in either of immediately frozen or 40-hour-incubated samples. The positions corresponding to molecular size markers are shown on the very left. The unincubated samples (0 hr) were normalized by their p24 concentrations (20 ng/well). 40hr incubated samples were applied as same volumes as each unincubated (0 hr) sample. Results shown are representatives of two (WB using MA antibody) or three (WB using Env antibody) independent experiments.

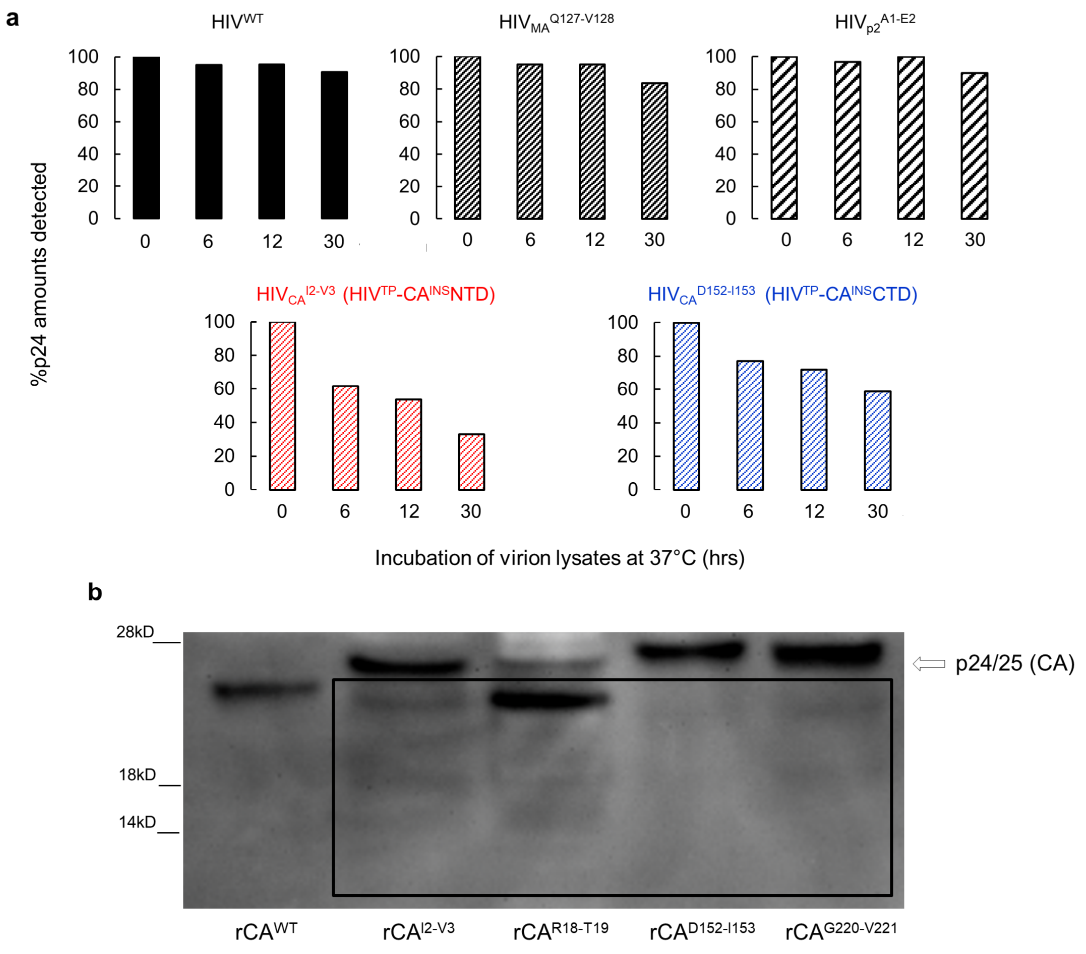


**Fig. S3. Time-Dependent Progression of the CA Degradation is Seen in only HIVs Containing an AA Insert in CA, and CA Degradation Products (Orderly CA Fragmentation) Observed in Individually Expressed CA$^{INS}$s.**

**(a)** The supernatants of HEK-293 cells producing HIV$^{WT}$ or HIV$^{TP}$ after transfection and 72-hour culture were filtered, ultracentrifuged, lysed, incubated for 0, 6, 12, or 30 hours at 37°C, and subjected to p24 ELISA. Each sample was immediately frozen at the end of the incubation and kept at -80°C until use. Note that the greatest time-dependent reduction was seen in the virion lysates with HIV$_{CA}^{I2\text{-}V3}$ (HIV$^{TP}$-CA$^{INS}$NTD), followed by the lysates with HIV$_{CA}^{D152\text{-}I153}$ (HIV$^{TP}$-CA$^{INS}$CTD). No reduction was seen with or without of incubation in the virion lysates with HIV$^{WT}$,

$HIV_{MA}^{Q127\text{-}V128}$, or $HIV_{p2}^{A1\text{-}E2}$. **(b)** COS-7 cells producing recombinant $HIV^{WT}$ CA ($rCA^{WT}$) or various recombinant CA containing the 19-AA insert at different positions (two recombinant $rCA^{INS}$NTD proteins: $rCA^{I2\text{-}V3}$ and $rCA^{R18\text{-}T19}$; two recombinant $rCA^{INS}$CTD proteins: $rCA^{D152\text{-}I153}$ and $rCA^{G220\text{-}V221}$), as shown in Fig 2, were lysed and the lysates were immediately (without incubation) subjected to WB with anti-p24 polyclonal anti-serum. Note the ladder-like degradates were observed in unincubated COS-7 cell lysates expressing $rCA^{INS}$NTDs and $rCA^{INS}$CTD. All samples were normalized by their p24 concentrations (5 ng/well). Results shown is representative of three independent experiments.

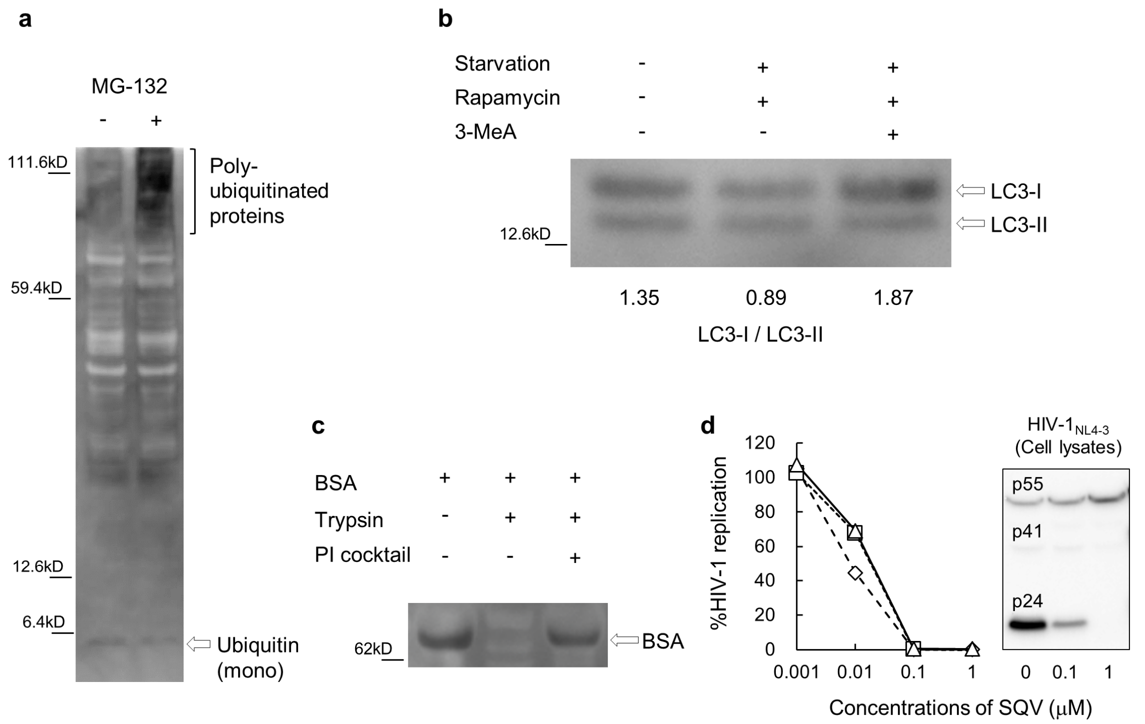


**Fig. S4. The CA Degradation Observed Has No Association with Cellular/viral Proteolytic Mechanisms.**

MG-132, an inhibitor of proteasome; 3-MeA, an autophagy inhibitor; a mixture of 7 potent cellular protease inhibitors; and SQV, a potent HIV-1-derived aspartyl protease failed to block CA degradation (Fig 3a-d). **(a)** Proteasome activity inhibition of MG-132 was confirmed by WB. Lane 2 indicated an accumulation of poly-ubiquitinated proteins in the MG-132-treated sample, which are physiologically decomposed and recycled by proteasome. If proteasome worked properly, the accumulation of poly-ubiquitinated proteins should not be detected. Both samples were normalized by their protein concentrations (20 μg/well). Results shown is representative of 2 independent experiments. **(b)** Changes in the ratio of LC3-I/LC3-II under 3-MeA treatment. Autophagosome forms its membrane by recruiting LC3-II, a truncated form of LC3-I, so that under the autophagy-inducing condition, a ratio of LC3-I/LC3-II should be decreased [46]. In Fig. 4b, a ratio of LC3-I/LC3-II decreased under the

autophagy-inducing condition (Strvation$^+$, Rapamycin$^+$; lane 2), while in the presence of 3-MeA, a ratio of LC3-I/LC3-II recovered (lane 3). Therefore, panels **(a)** and **(b)** denote that treatments of MG-132 and 3-MeA properly inhibited the function of corresponding cellular protein degradation systems, ubiquitin/proteasome and autophagy, respectively. All samples were normalized by their protein concentrations (30 μg/well). Results shown is representative of 5 independent experiments. **(c)** The cocktail of 7 cellular PIs completely blocked the digestion of bovine serum albumin by trypsin. All samples were normalized by their protein concentrations (10 μg/well). Results shown is representative of 2 independent experiments. **(d)** (left panel) Inhibitory activity of SQV was confirmed to potently block HIV-1 replication in the HIV-1 replication suppression assay as assessed using the MTT assay employing HIV-$1_{LAI}$ and MT-2 cells [47, 48]. Results shown were obtained from three independent experiments. (right panel) WB image representing the inhibitory effect of Gag processing by SQV. COS-7 cells were transfected with pHIV-$1_{NL4-3}$ with different concentration of SQV in the culture supernatants. WB using the lysates of COS-7 cells expressing HIV-$1_{NL4-3}$ and anti-p24 polyclonal anti-serum clearly showed that the cleavage of Gag protein was inhibited by the addition of SQV. Results shown is representative of 10 independent experiments.

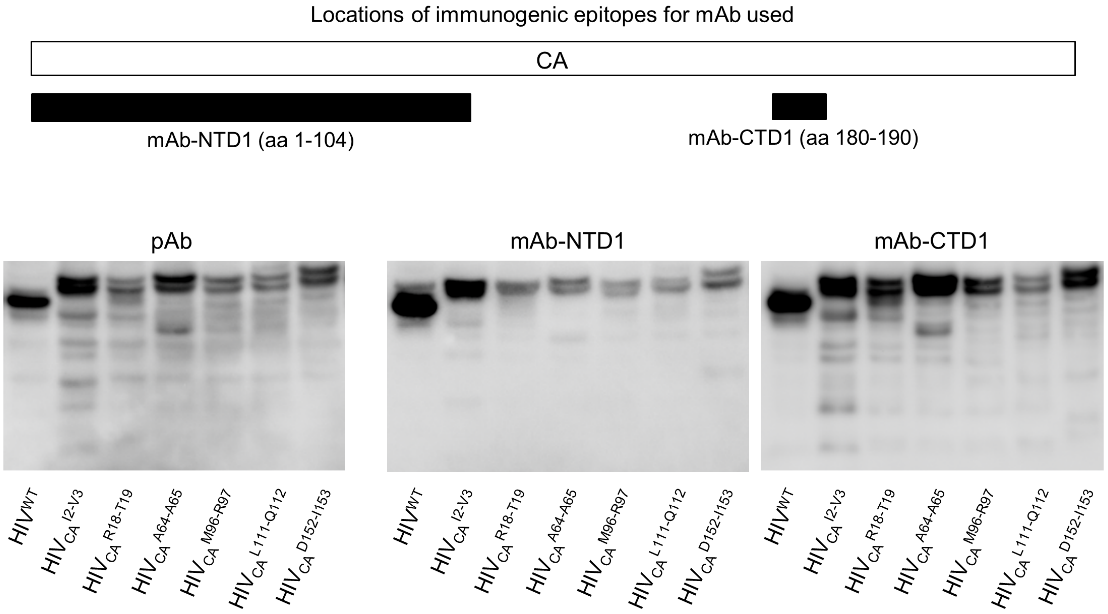


**Fig. S5. Identification of the Region Responsible for the Initiation of CA Degradation.**

Two different monoclonal antibodies (mAbs) that recognize different antigenic epitopes of CA were employed to identify the responsible region for the CA degradation. The lysates of HEK-293 cells producing $HIV^{WT}$ or 6 $HIV^{TP}_{CA}$ ($HIV_{CA}^{I2\text{-}V3}$, $HIV_{CA}^{R18\text{-}T19}$, $HIV_{CA}^{A64\text{-}A65}$, $HIV_{CA}^{M96\text{-}R97}$, $HIV_{CA}^{L111\text{-}Q112}$, and $HIV_{CA}^{D152\text{-}I153}$) were subjected to WB using mAb-NTD1 and mAb-CTD1. The locations of each immunogen recognized are shown in the top. Note that large amounts of CA degradates were seen as examined with polyclonal anti-CA anti-serum and mAb-CTD1, while virtually no degradates were identified as examined with mAb-NTD1. All samples were normalized by their protein concentrations (20 µg/well). Results shown is representative of two independent experiments for each mAb.

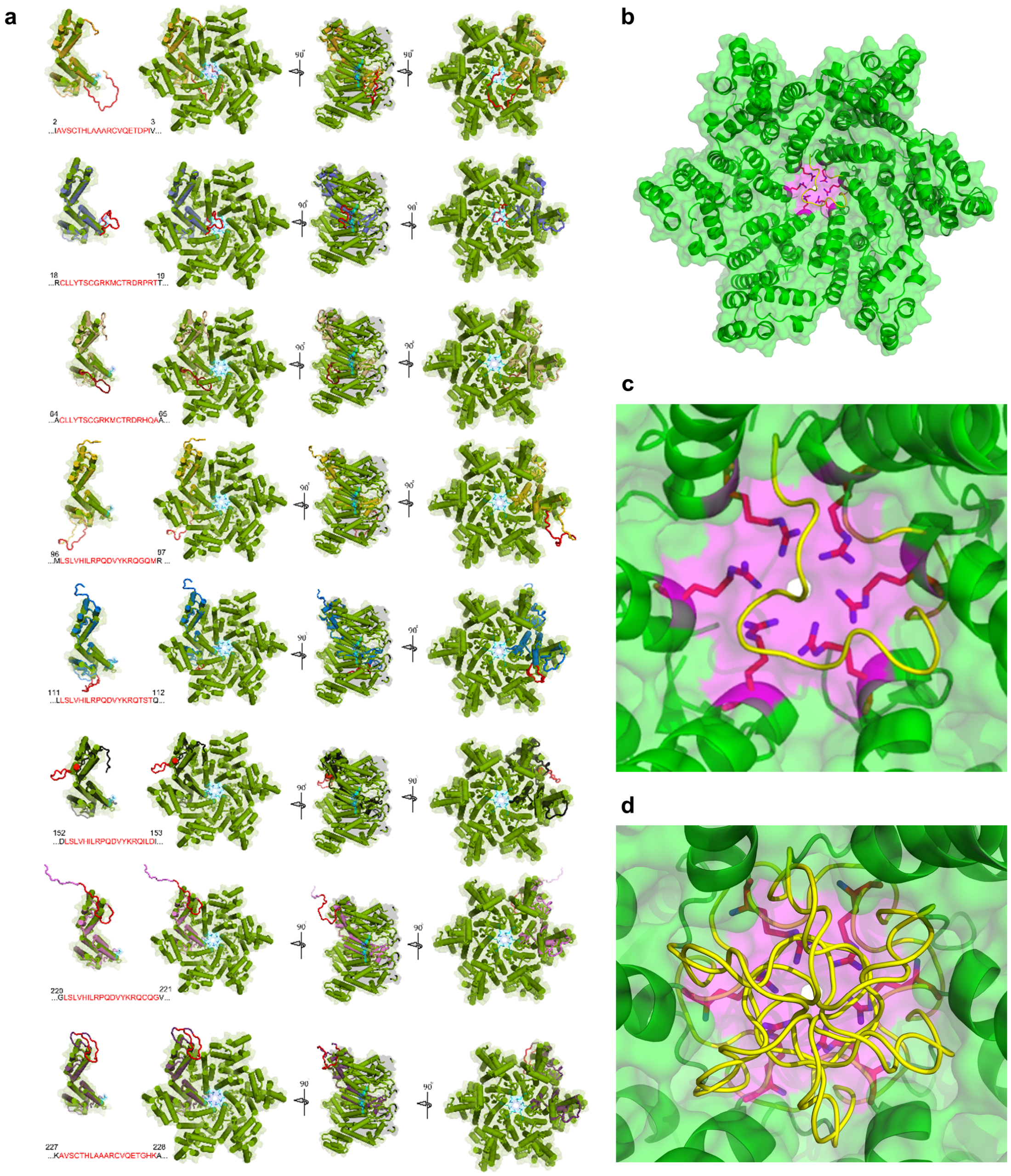


**Fig. S6. Structural Modeling of rCA Variant-Superimposed CA$^{WT}$ Monomer, and Structural Alterations of CA Hexamer Caused by the 19-AA Insertion, and Structure of a CA Hexamer, Comprised of 6 CA$^{I2-V3}$s, All of which Contain the 19-AA Insert.**

**(a)** Homology models of CA monomer with insertions of A64-A65, M96-R97,

L111-Q112, D152-I153, G220-V221, and K227-A228 were generated using a structure prediction server (PS)[2] version 3.0. Note that in $CA^{A64-A65}$, $CA^{M96-R97}$, $CA^{L111-Q112}$, $CA^{D152-I153}$, $CA^{G220-V221}$, $CA^{K227-A228}$, the 19-AA insertion occurred in the proximity of H4, H5, H6, H7, and H11, respectively, and each insert is located relatively distant form the center of the hexamer and seems to cause less drastic effects on the structure of the hexamer and the pore. In order to make the location of each insert clear, only one insert is shown in the hexamer. **(b)** The 19-AA insert in CA monomer is indicated in yellow in $CA^{I2-V3}$ hexamer. **(c)** A magnified image of one insert in CA monomer on $CA^{I2-V3}$ hexamer, which seems to directly interfere with the structure and integrity of the CA pore. **(d)** A magnified image of 6 inserts in $CA^{I2-V3}$ hexamer. Note that the presence of six 19-AA inserts leads to the formation of extended loops, which wrap around the CA pore composed of 6 arginine residues (shown in cyan). It is assumed that the extended loops highly likely push each monomer apart and effectively block the formation of physiologic hexamer complexes.

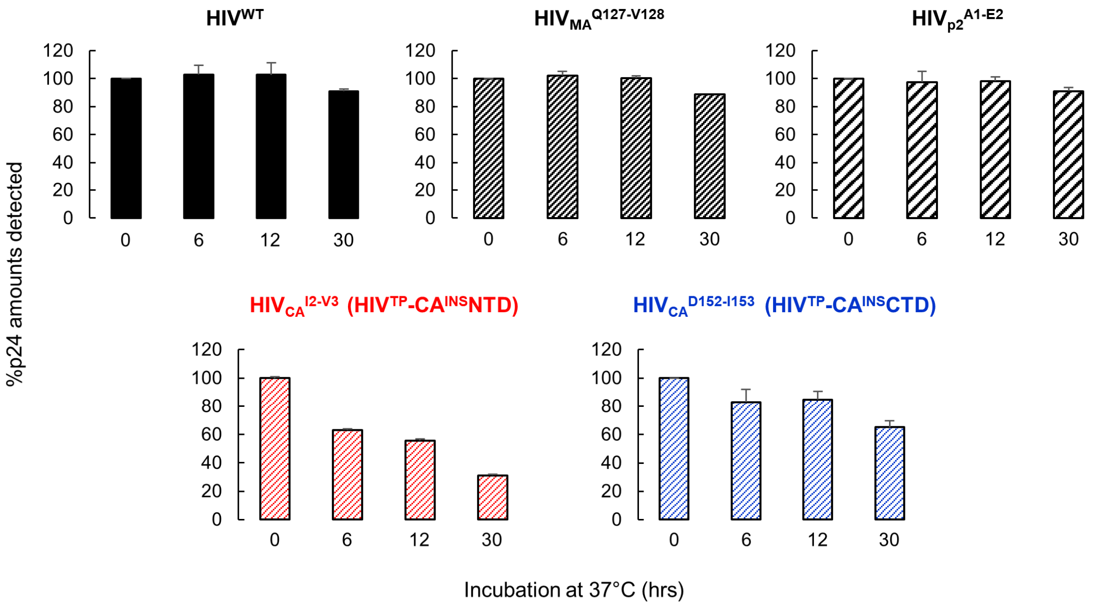


**Fig. S7. Time-Dependent Progression of CA Degradation Examined with the RETROtek™ ELISA System.**

The supernatants of transfected HEK-293 cells producing $HIV^{WT}$ or $HIV^{TP}$s were collected 72 hours after transfection, ultracentrifuged (20,000 *g*, 4°C, O/N), and lysed with the lysing buffer. Each virion lysates preparation was incubated at 37°C for different periods of time and examined with RETROtek™ ELISA that employs a CA-specific monoclonal antibody different from the CA-specific mAb of Lumipulse *f*. Note that the immunogenicity was lose time-dependently with the use of RETROtek™ ELISA as examined with Lumipulse *f*. Data are represented as mean ± S.D. (n=2).

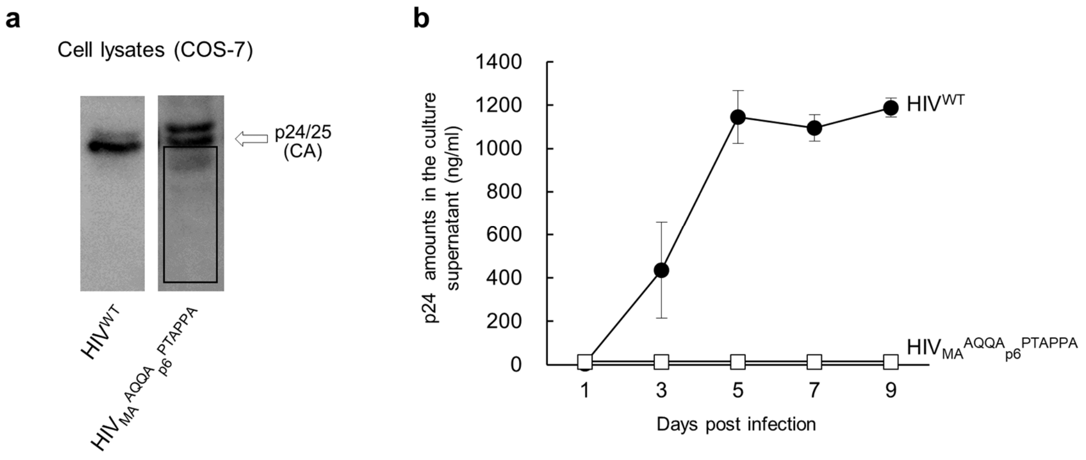


**Fig. S8. CA degradation and reduced replicability observed in HIV$_{MA}$$^{AQQA}$$_{p6}$$^{PTAPPA}$.**

**(a)** CA degradation in the lysates of COS-7 cells producing HIV$_{MA}$$^{AQQA}$$_{p6}$$^{PTAPPA}$. The lysates of COS-7 cells producing HIV$^{WT}$ or HIV$_{MA}$$^{AQQA}$$_{p6}$$^{PTAPPA}$, which contains AQQA and PTAPPA inserts in Gag-encoding region that did not cause "severe' reduction in viral replication in our previous report [19] with wild-type PR, were examined for enhanced degradation. Each lysate sample was prepared 72 hours after transfection and subjected to WB with anti-p24 polyclonal anti-serum. All samples were normalized by their protein concentrations (20 µg/well). **(b)** Loss of replication capacity of HIV$_{MA}$$^{AQQA}$$_{p6}$$^{PTAPPA}$. Replication kinetics was determined with the amounts of p24 production in cultures of MT-4 cells exposed to each HIV$^{WT}$ or HIV$_{MA}$$^{AQQA}$$_{p6}$$^{PTAPPA}$. All p24 values are single-point determinations. Results indicate that HIV$_{MA}$$^{AQQA}$$_{p6}$$^{PTAPPA}$ examined completely failed to replicate in MT-4 cells.

**Movie S1. Structure of a CA hexamer, comprised of one $CA^{I2\text{-}V3}$ and 5 $CA^{WT}$s.**

The movie shows a single HIV CA monomer model (shown in yellow) containing a single insertion between I2-V3 (in red) superimposed onto the hexamer CA structure without an insert (in green). The view zoomed in and out around the center pore region consisting of 6 Arg residues. The extended loop wraps around the pore region of the hexamer, which would effectively push monomers apart and thereby blocks hexamer assembly. The movie was made using a USCF Chimera and the hexameric CA structure derived from PDB ID: 5HGL.

## References for Supplemental materials


46. Kabeya, Y. et al. LC3, a mammalian homologue of yeast Apg8p, is localized in autophagosome membranes after processing. *EMBO J.*, **19**, 5720–5728 (2000).

47. Salcedo-Gómez, P. M. et al. GRL-04810 and GRL-05010, Difluoride-Containing Nonpeptidic HIV-1 Protease Inhibitors (PIs) That Inhibit the Replication of MultiPI-Resistant HIV-1 In Vitro and Possess Favorable Lipophilicity That May Allow Blood-Brain Barrier Penetration. *Antimicrob. Agents Chemother.*, **57**, 6110–6121 (2013).

48. Amano, M. et al. GRL-09510, a Unique P2-Crown-Tetrahydrofuranylurethane-Containing HIV-1 Protease Inhibitor, Maintains Its Favorable Antiviral Activity against Highly-Drug-Resistant HIV-1 Variants in vitro. *Sci. Rep.*, **7**, 12235 (2017).

**Table S1. AA Sequences of the Inserts Examined in This Study.**

Three codons were randomly changed in the insertion using the EZ-Tn5 Transposon system,

http://www.lucigen.com/docs/slide-decks/Lucigen-EZ-Tn5-Transposon-Tools.pdf

| $HIV^{TP}$s studied | AA sequences of an insert |
|---|---|
| $HIV_{MA}^{V35\text{-}W36}$ | LSLVHILRPQDVYKRQHIV |
| $HIV_{MA}^{G62\text{-}Q63}$ | AVSCTHLAAARCVQETVLG |
| $HIV_{MA}^{V88\text{-}H89}$ | AVSCTHLAAARCVQETDCV |
| $HIV_{MA}^{E105\text{-}E106}$ | AVSCTHLAAARCVQETEIE |
| $HIV_{MA}^{Q127\text{-}V128}$ | AVSCTHLAAARCVQETGNQ |
| $HIV_{CA}^{I2\text{-}V3}$ | AVSCTHLAAARCVQETDPI |
| $HIV_{CA}^{R18\text{-}T19}$ | CLLYTSCGRKMCTRDRPRT |
| $HIV_{CA}^{A64\text{-}A65}$ | CLLYTSCGRKMCTRDRHQA |
| $HIV_{CA}^{M96\text{-}R97}$ | LSLVHILRPQDVYKRQGQM |
| $HIV_{CA}^{L111\text{-}Q112}$ | LSLVHILRPQDVYKRQTST |
| $HIV_{CA}^{D152\text{-}I153}$ | LSLVHILRPQDVYKRQILD |
| $HIV_{CA}^{G220\text{-}V221}$ | LSLVHILRPQDVYKRQCQG |
| $HIV_{CA}^{K227\text{-}A228}$ | AVSCTHLAAARCVQETGHK |
| $HIV_{p2}^{A1\text{-}E2}$ | CLLYTSCGRKMCTRDRVLA |
| $HIV_{p6}^{T8\text{-}A9}$ | LSLVHILRPQDVYKRQEPT |

**Table S2. Abnormal Degradation Observed in Individually Expressed CA Protein with the AA Insertion.**

| | p24 concentrations in the cell lysates (ng/ml) | | | | |
|---|---|---|---|---|---|
| incubation time | 0h | 6h | 12h | 24h | 48h |
| $rCA^{WT}$ | 5892.5 | 5823.1 | n.d. | 5078.6 | 4371.4 |
| $rCA^{I2\text{-}V3}$ | 1303.7 | 949.5 | 698.6 | 231.2 | 162.6 |
| $rCA^{R18\text{-}T19}$ | 550.7 | 400.3 | 311.5 | 149.4 | 75.7 |
| $rCA^{D152\text{-}I153}$ | 3648.2 | 3172.3 | n.d. | 1477.5 | 970.1 |
| $rCA^{G220\text{-}V221}$ | 1693.7 | 1412.0 | n.d. | 703.0 | 275.6 |

**Fig. S9. Full Unedited Blots/gels Images.**

Full unedited images for Figure 1.

**b**

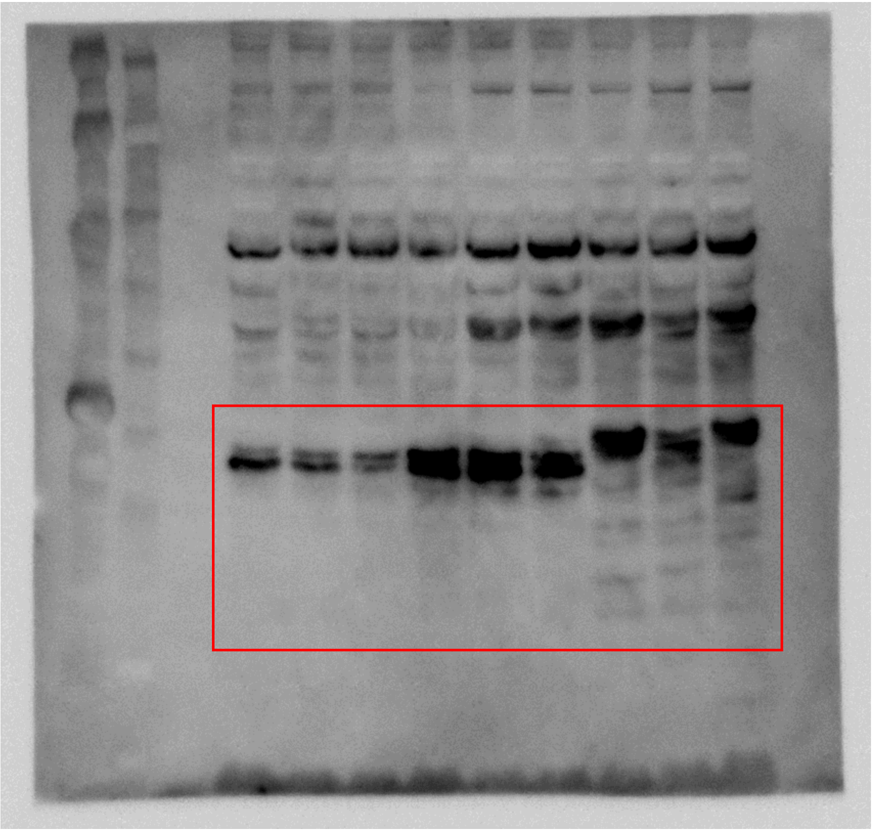

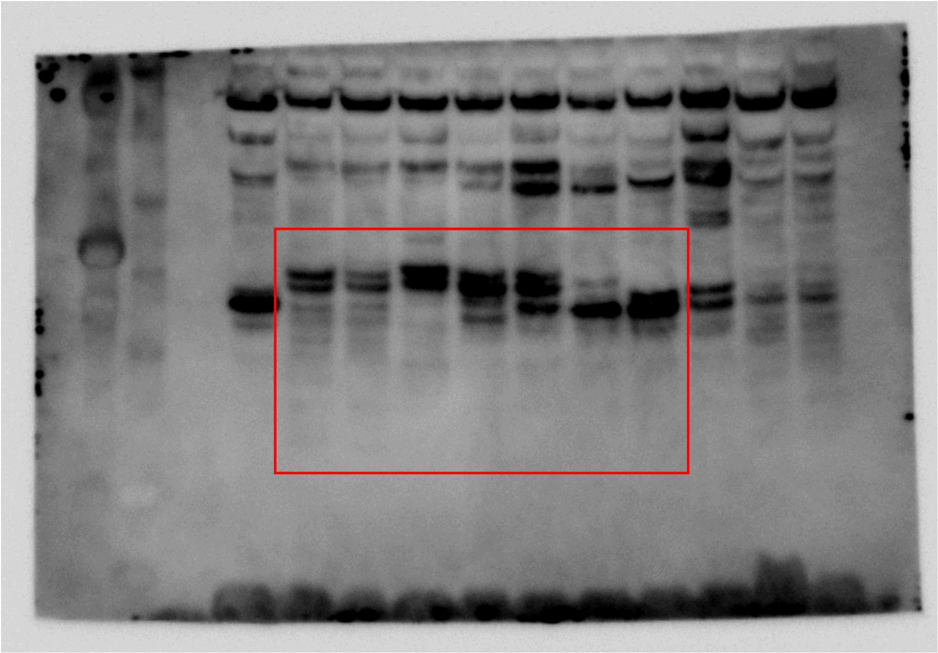

**Fig. S9. Full Unedited Blots/gels Images.**

Full unedited image for Figure 1.

c

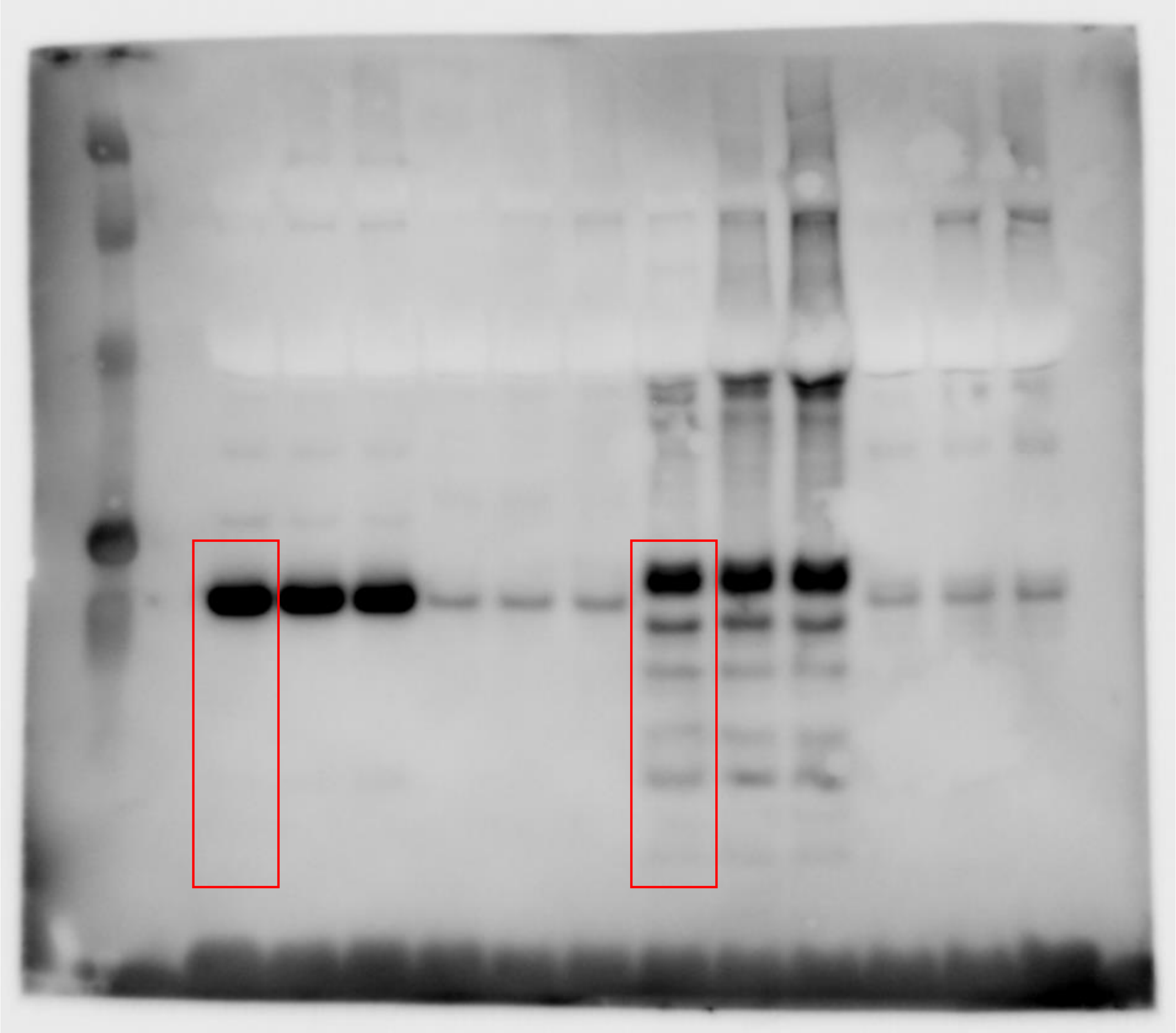

**Fig. S9. Full Unedited Blots/gels Images.**

Full unedited image for Figure 1.

d

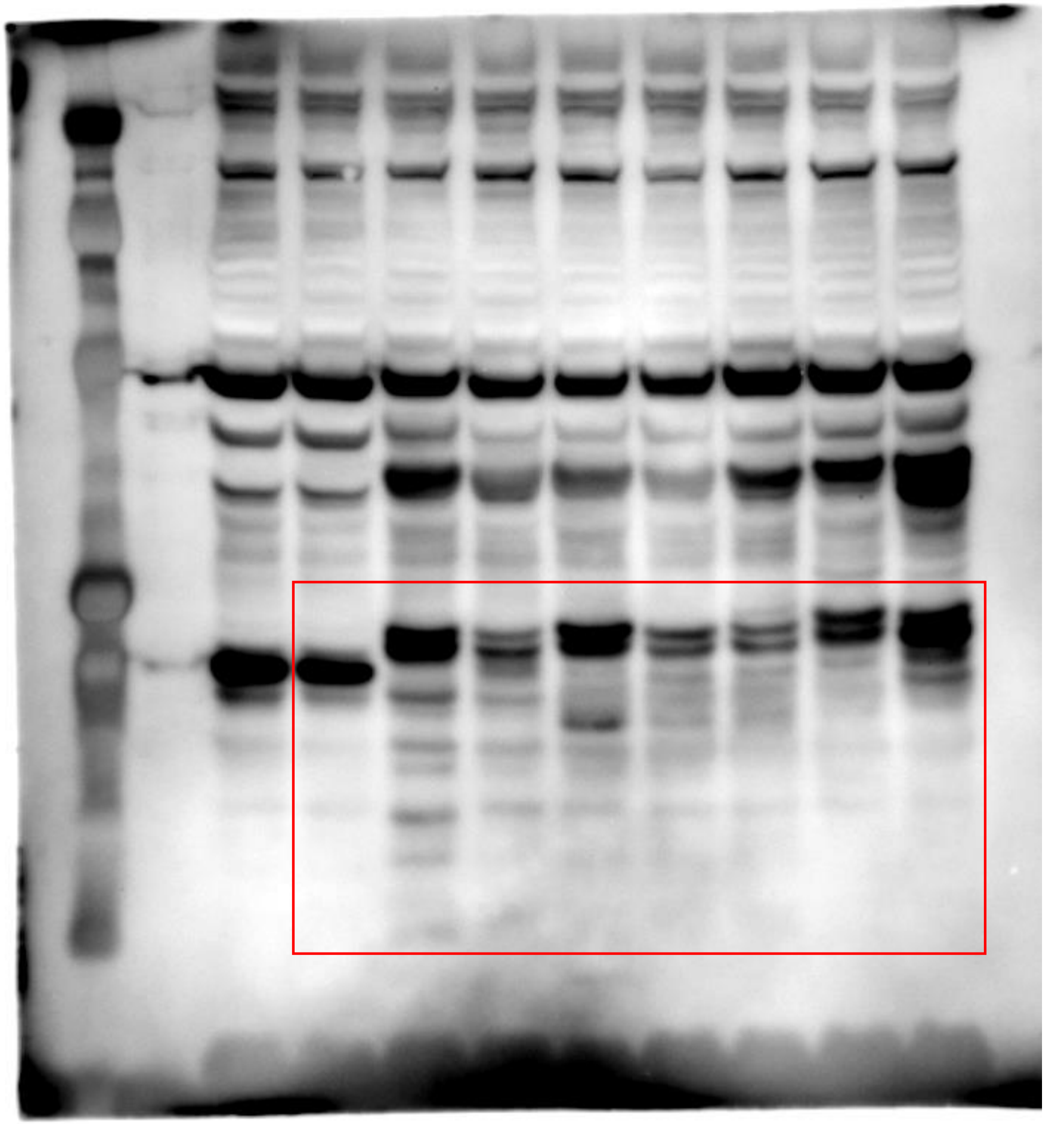

**Fig. S9. Full Unedited Blots/gels Images.**

Full unedited image for Figure 2.

c

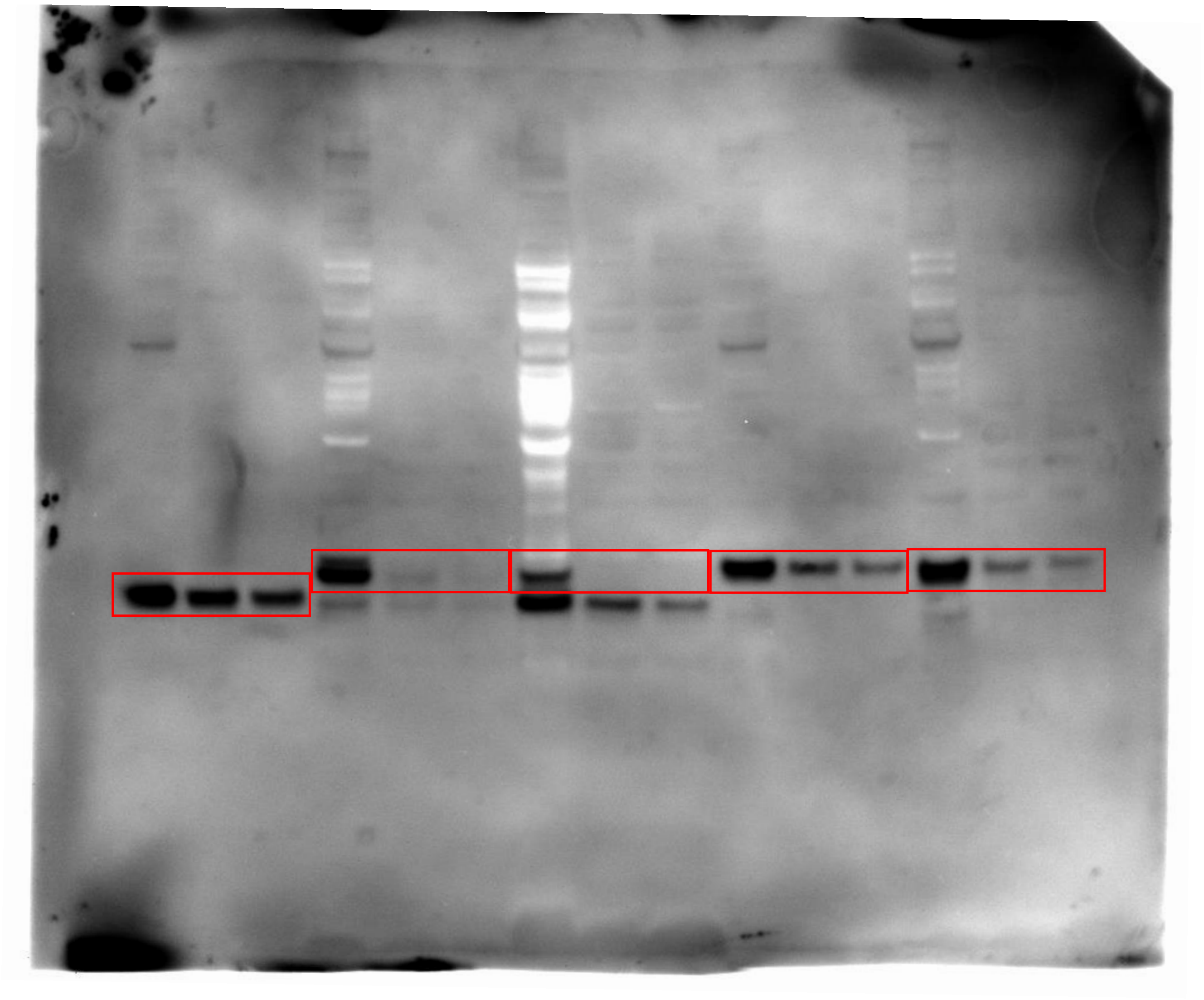

**Fig. S9. Full Unedited Blots/gels Images.**

Full unedited image for Figure 3.

a

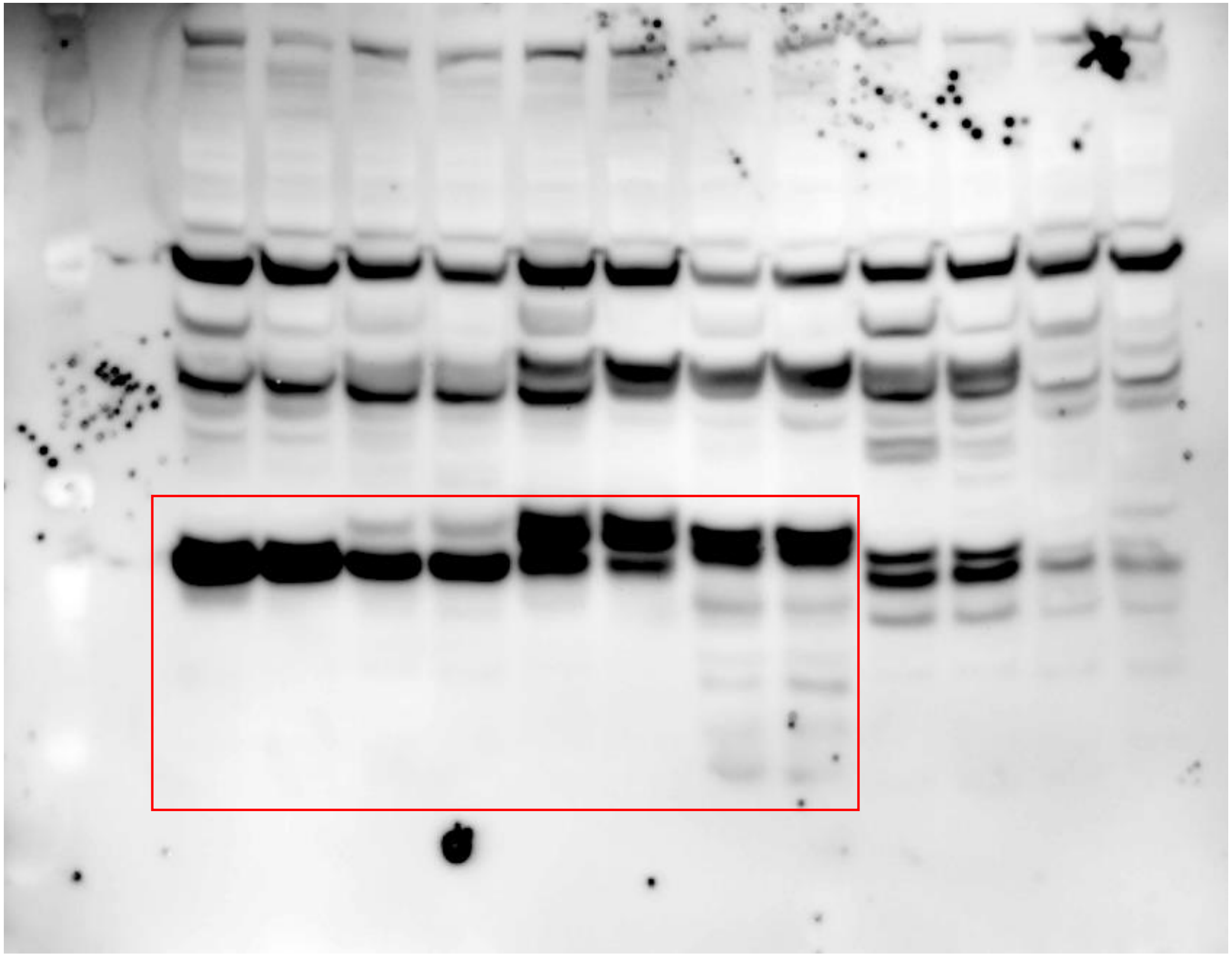

**Fig. S9. Full Unedited Blots/gels Images.**

Full unedited image for Figure 3.

b

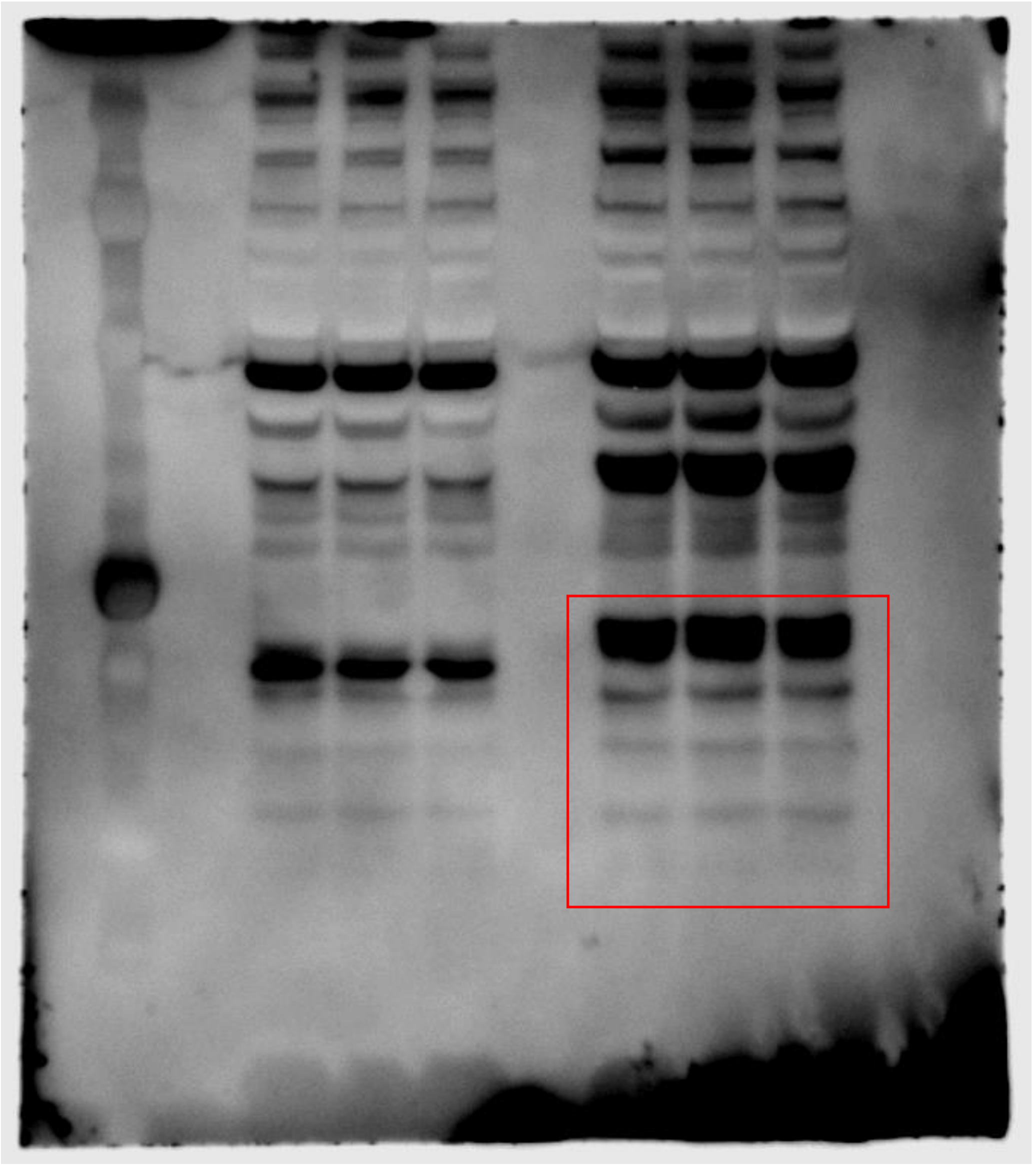

**Fig. S9. Full Unedited Blots/gels Images.**

## Full unedited images for Figure 3.

**f**

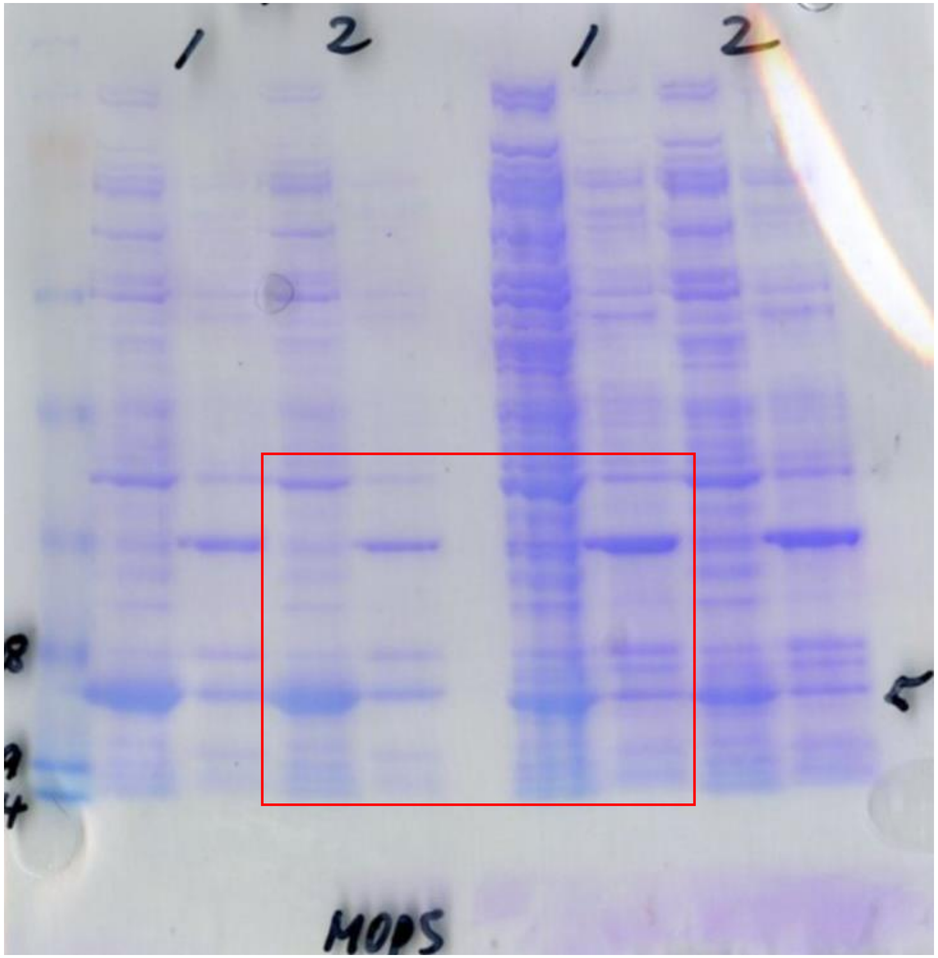


**g**

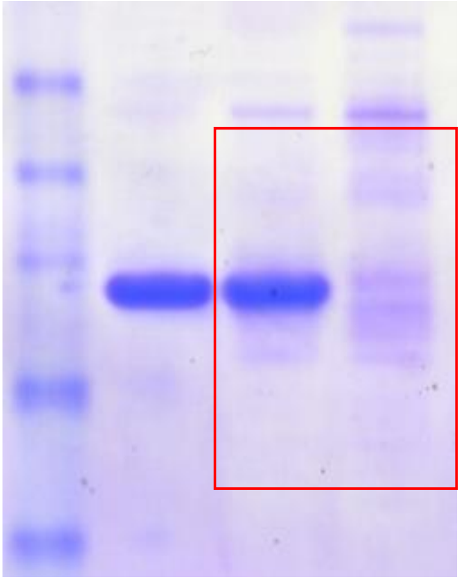

**Fig. S9. Full Unedited Blots/gels Images.**

Full unedited image for Figure 4.

**b**

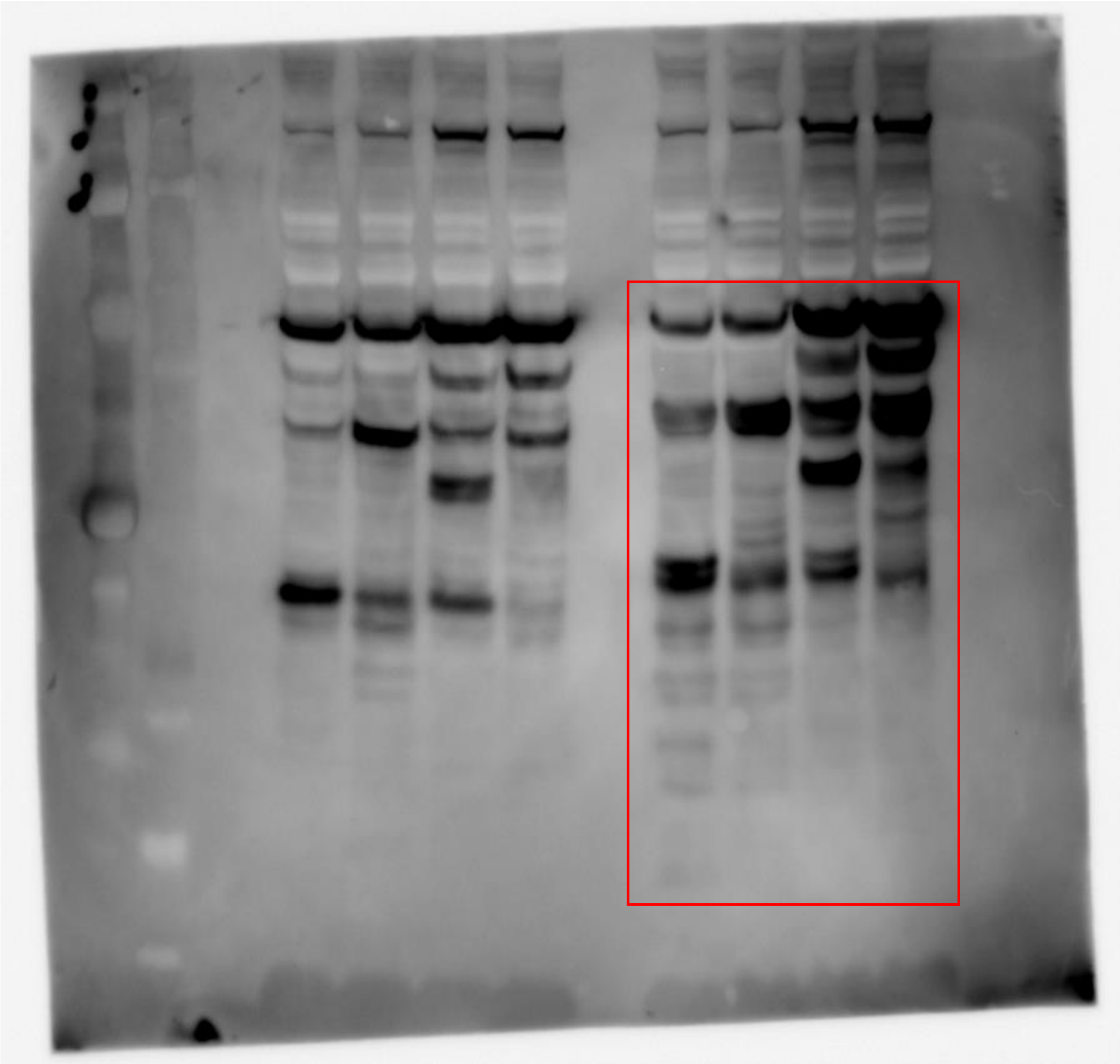

**Fig. S9. Full Unedited Blots/gels Images.**

Full unedited images for Figure 4.

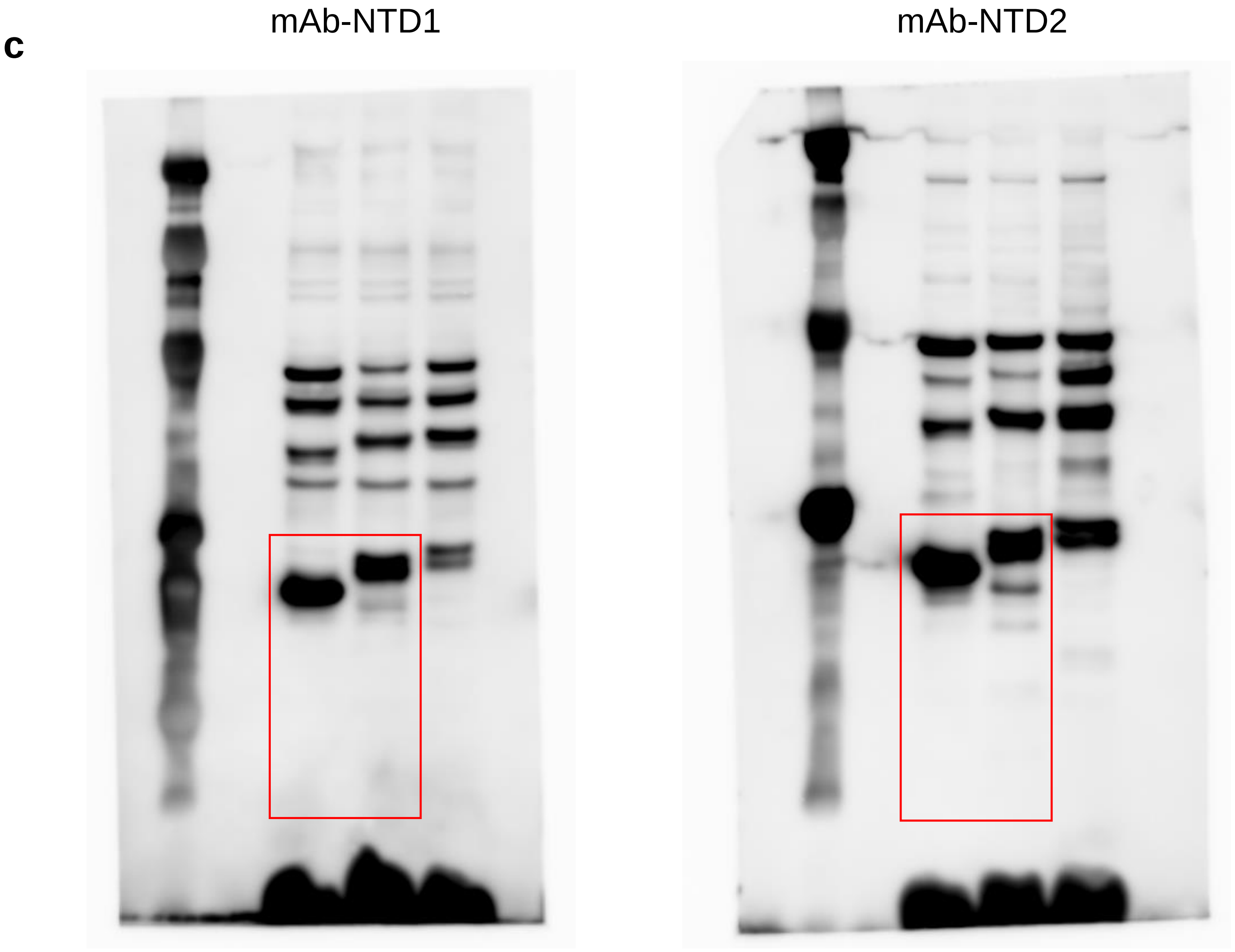

**Fig. S9. Full Unedited Blots/gels Images.**

Full unedited images for Figure 4.

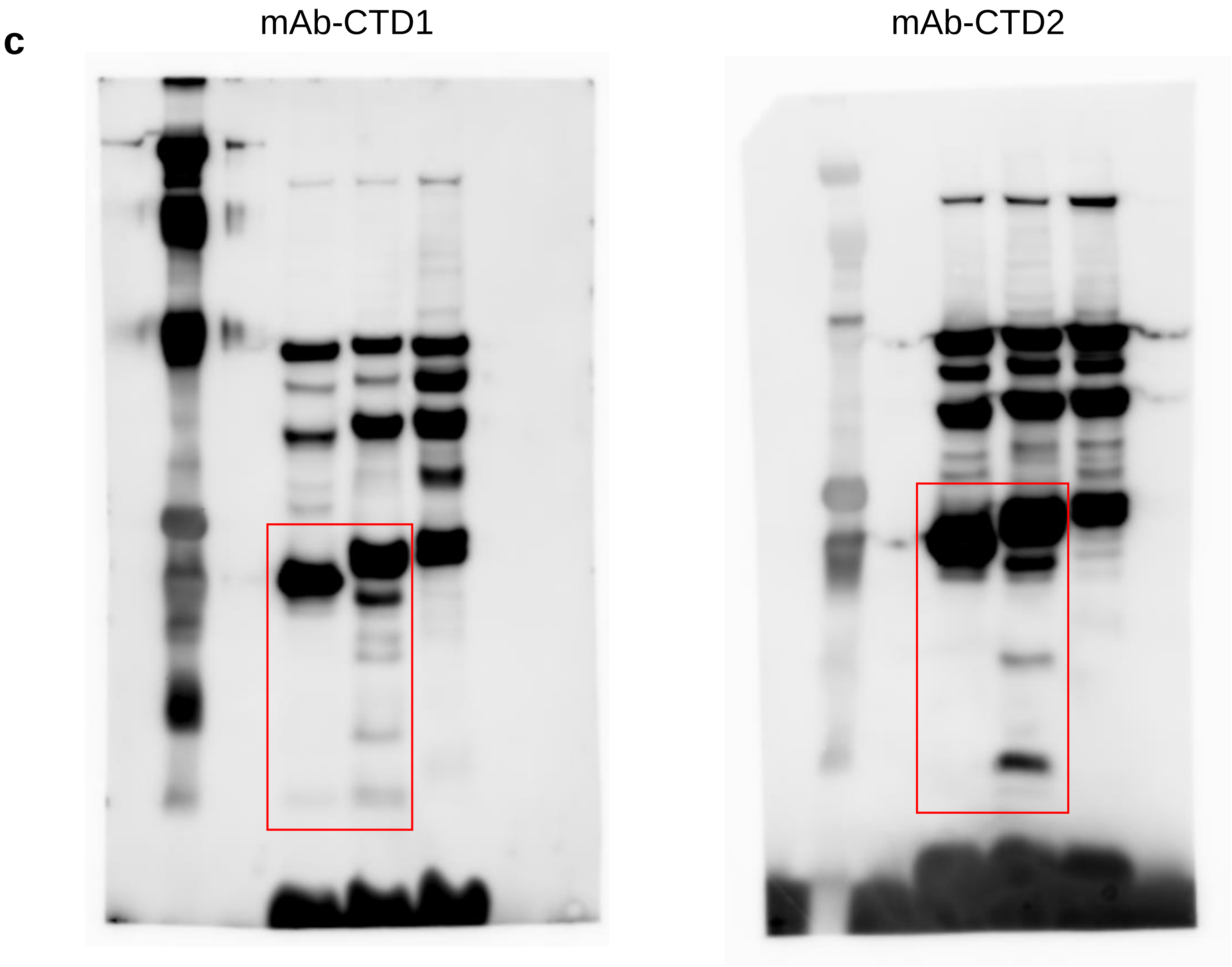

**Fig. S9. Full Unedited Blots/gels Images.**

Full unedited image for Figure S2.

**a**

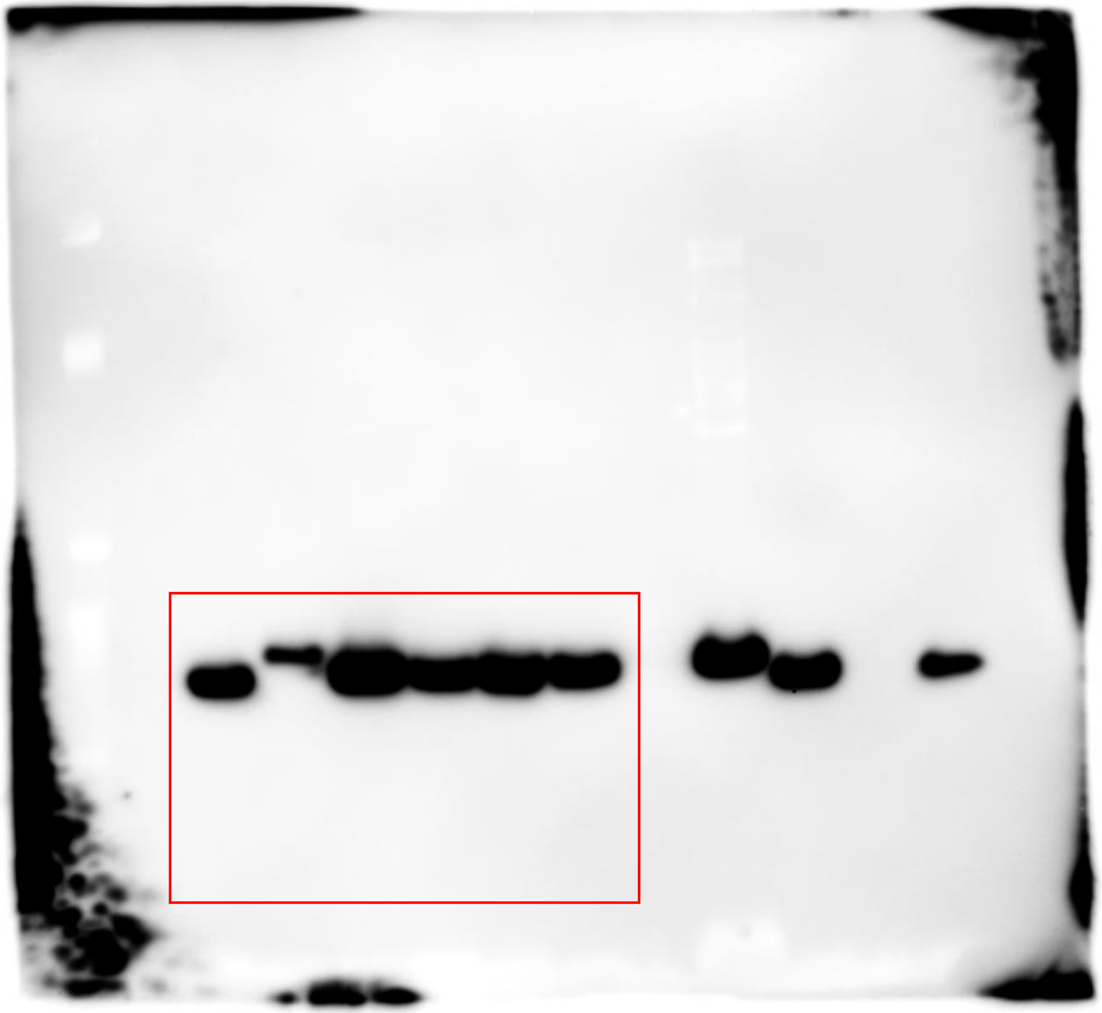

**Fig. S9. Full Unedited Blots/gels Images.**

Full unedited image for Figure S2.

**b**

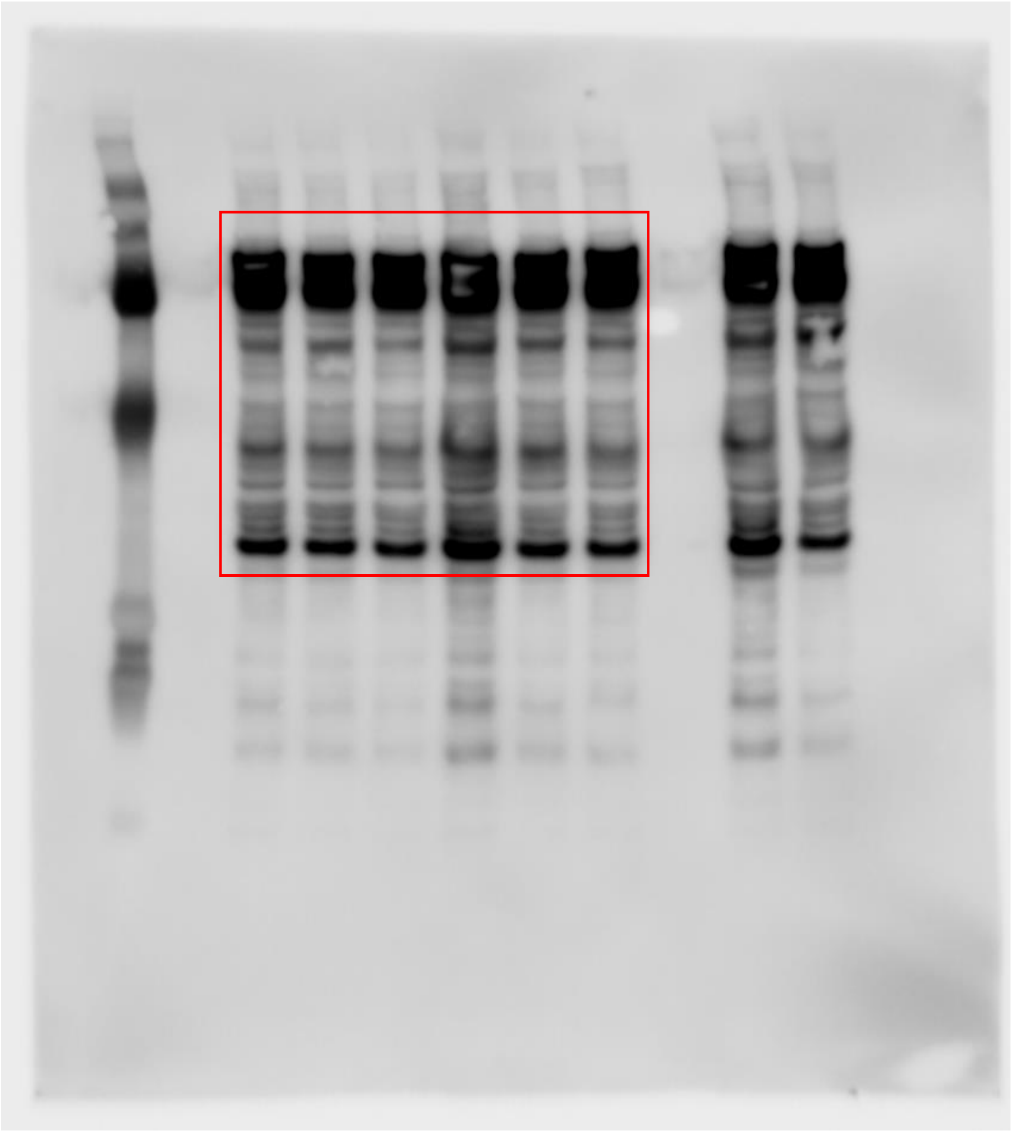

**Fig. S9. Full Unedited Blots/gels Images.**

Full unedited image for Figure S2.

c

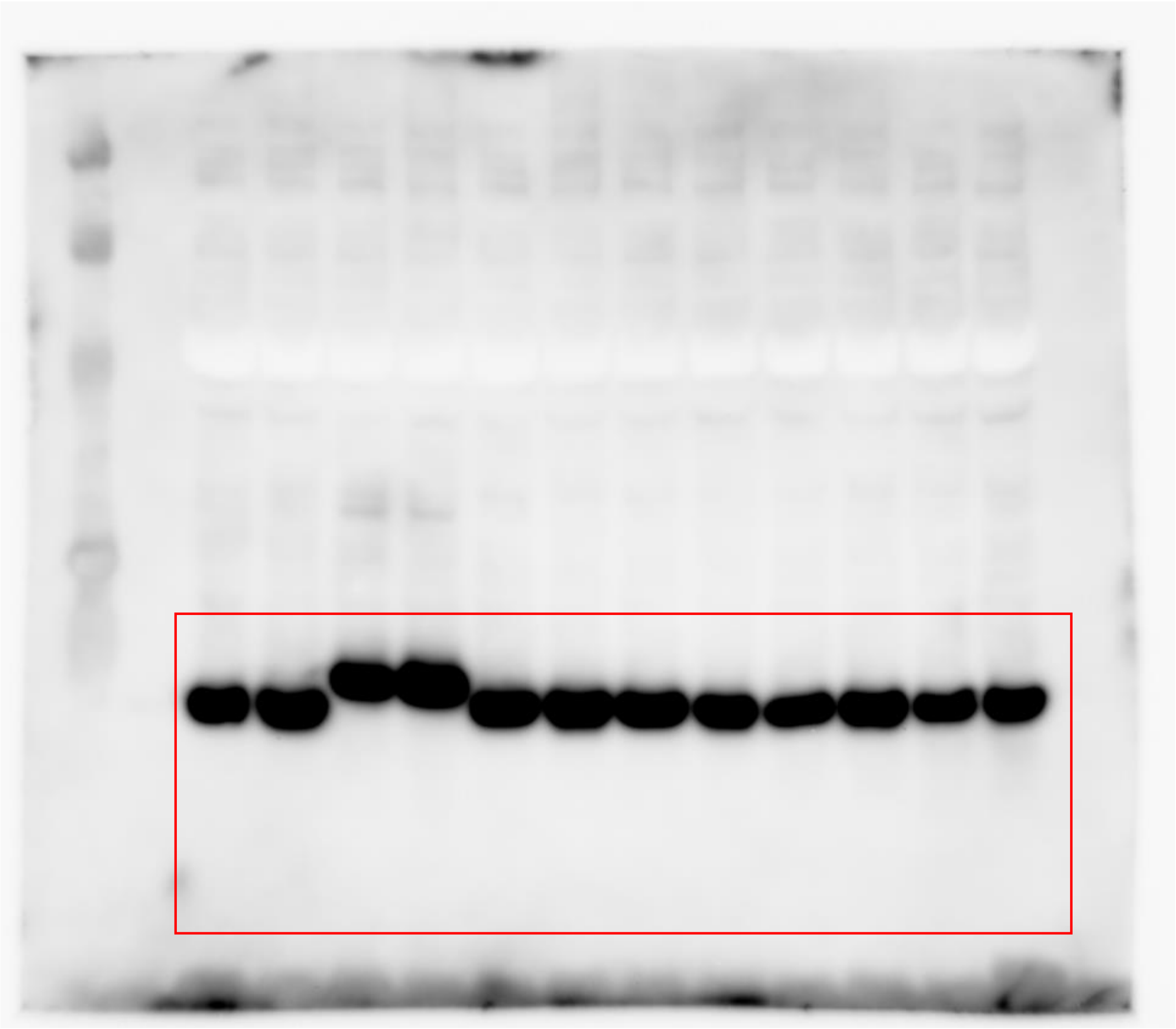

**Fig. S9. Full Unedited Blots/gels Images.**

Full unedited image for Figure S4.

a

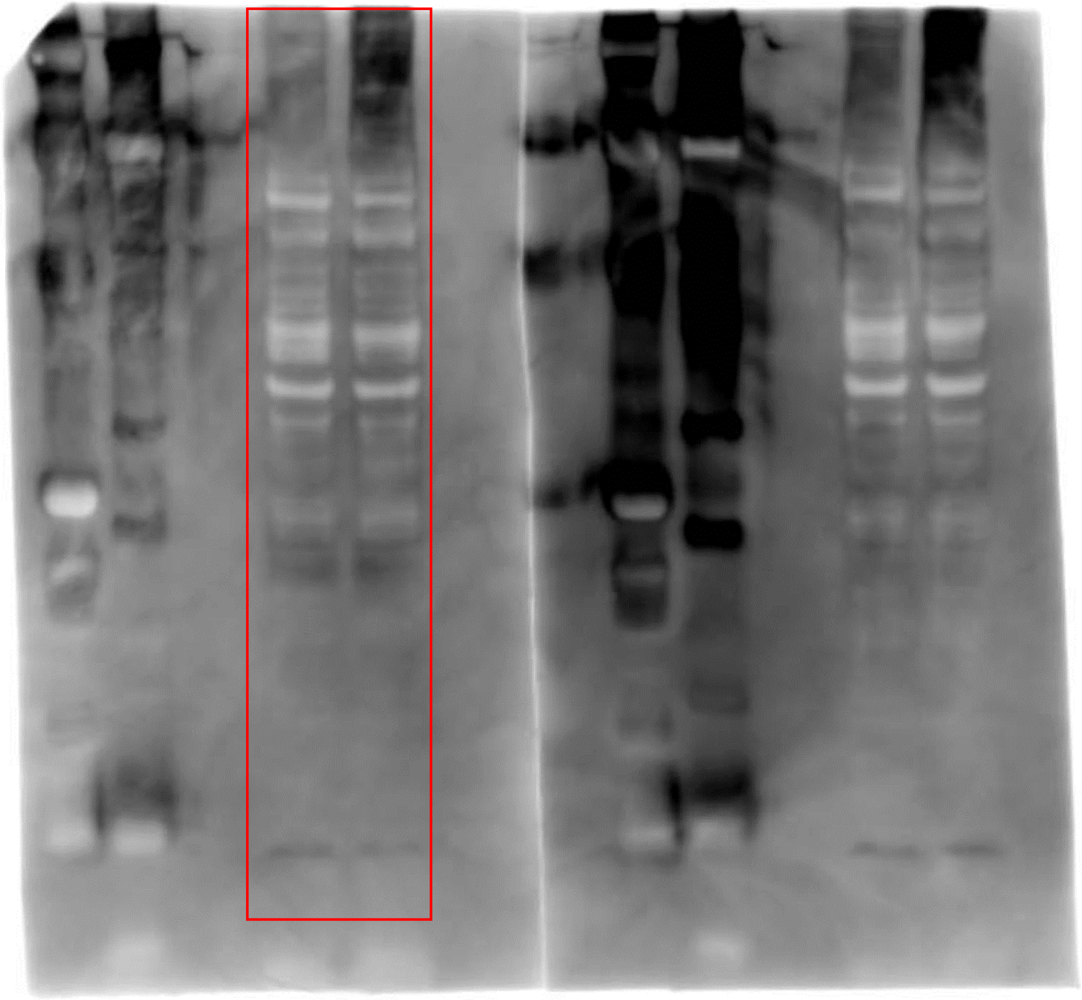

**Fig. S9. Full Unedited Blots/gels Images.**

Full unedited image for Figure S4.

**b**

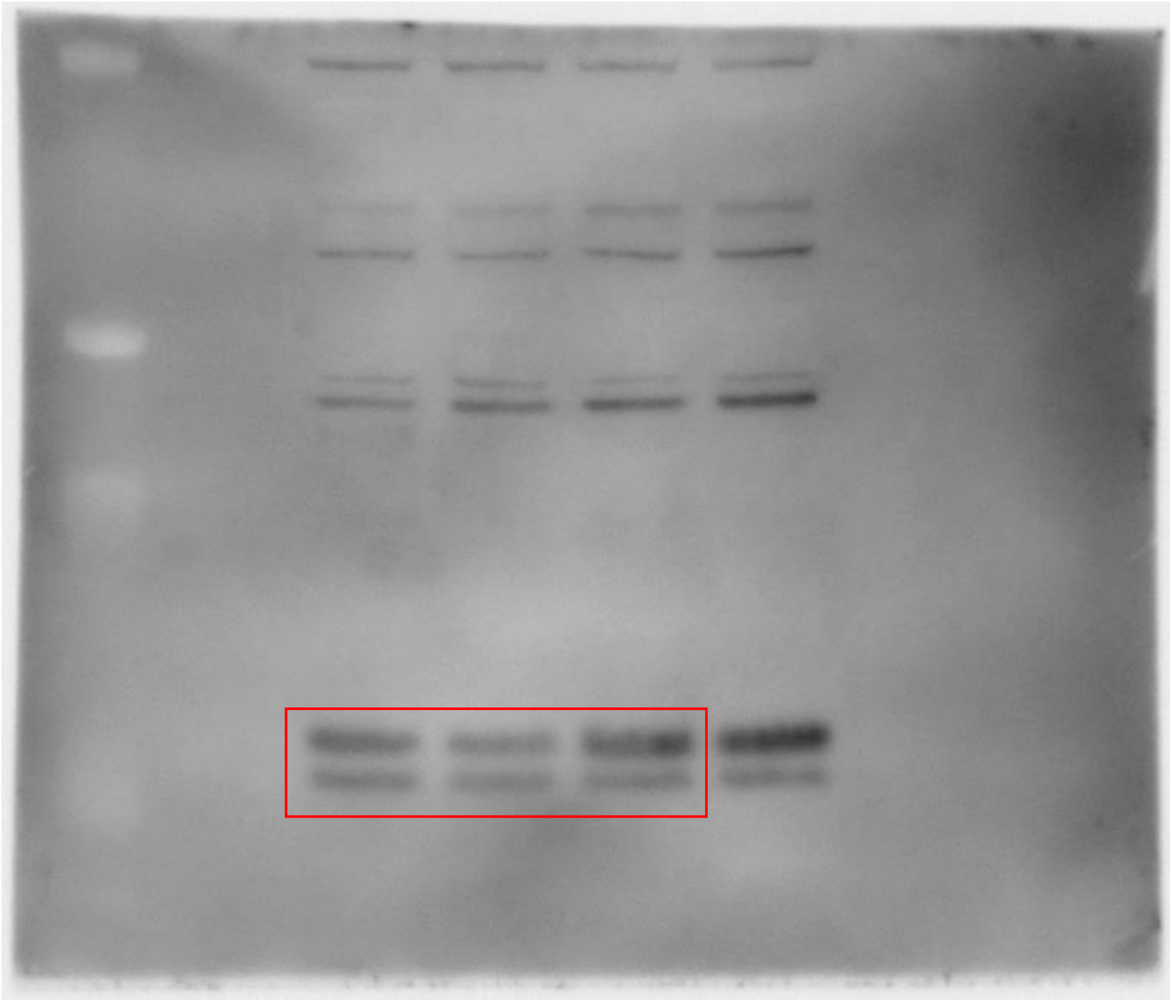

**Fig. S9. Full Unedited Blots/gels Images.**

Full unedited image for Figure S4.

c

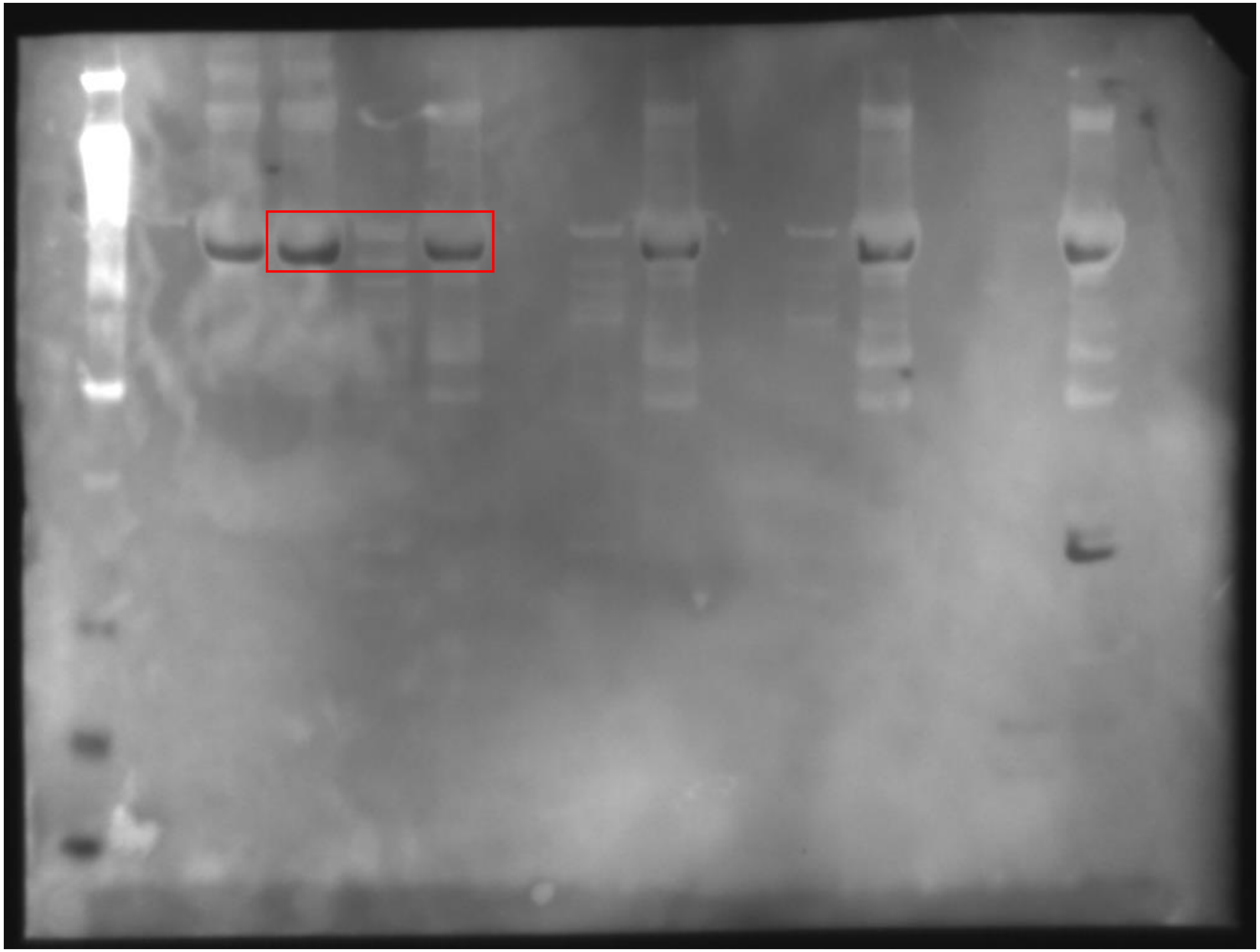

**Fig. S9. Full Unedited Blots/gels Images.**

Full unedited image for Figure S5.

mAb-NTD1

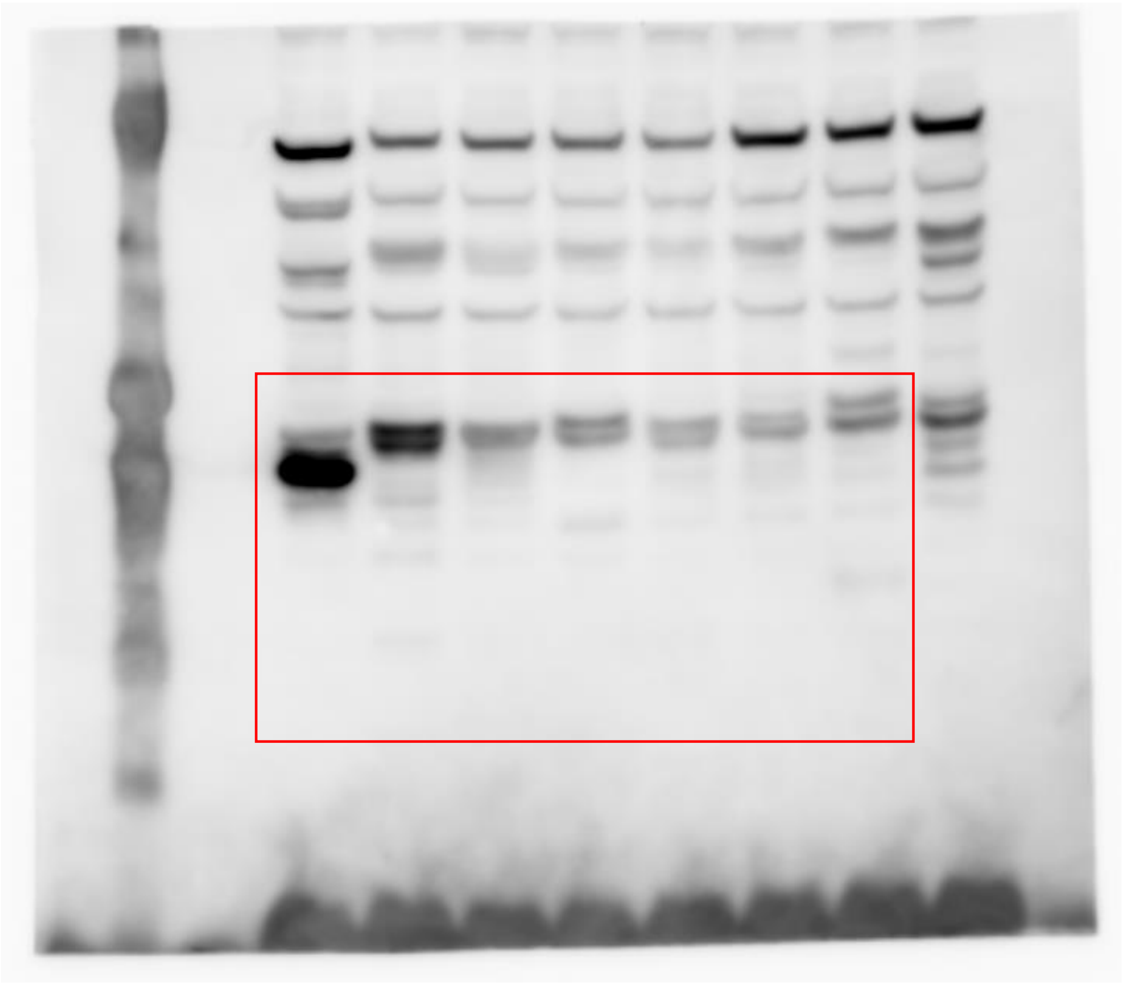

**Fig. S9. Full Unedited Blots/gels Images.**

Full unedited image for Figure S5.

mAb-CTD1

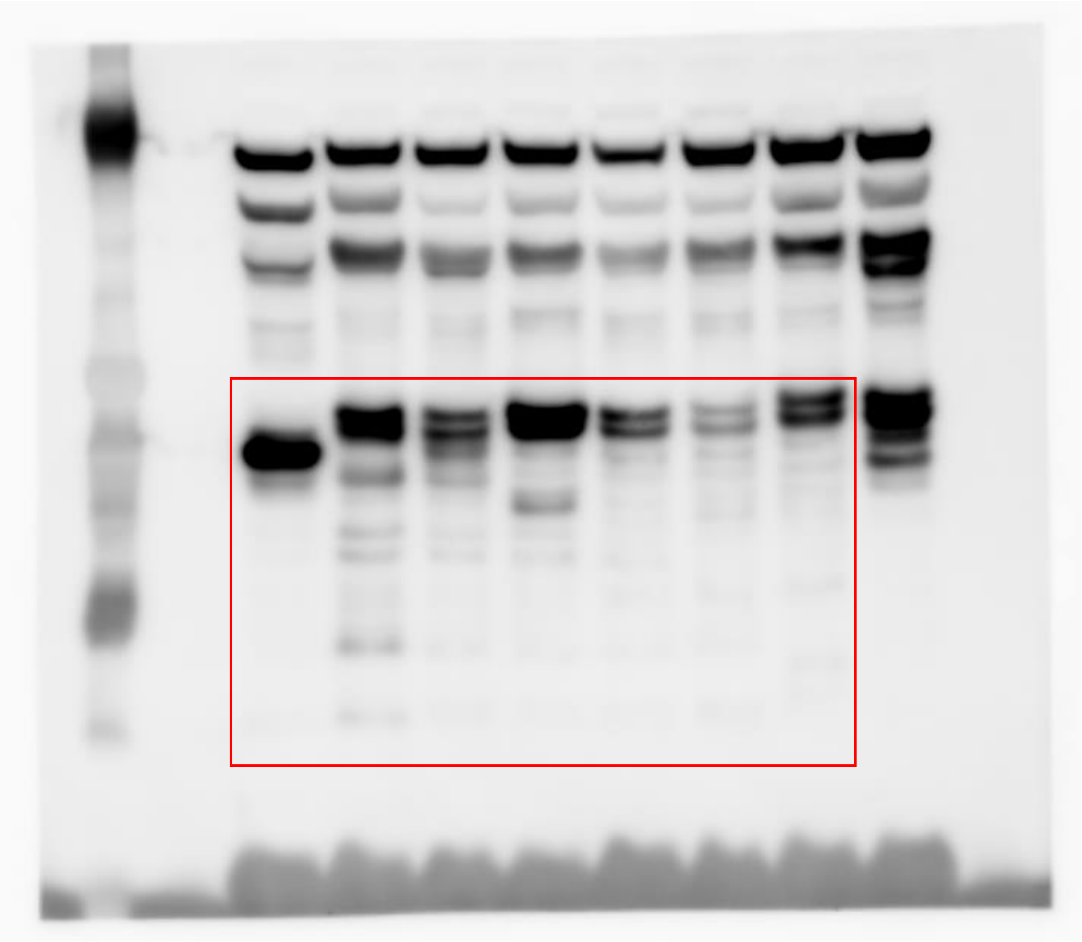

Supplement: Supplementary file 1 — Supplementary materials [file 41598_2019_46082_MOESM1_ESM.pdf]
